# Characterization of Cell Wall Lipids from the Pathogenic Phase of *Paracoccidioides brasiliensis* Cultivated in the Presence or Absence of Human Plasma

**DOI:** 10.1371/journal.pone.0063372

**Published:** 2013-05-17

**Authors:** Larissa V. G. Longo, Ernesto S. Nakayasu, Felipe Gazos-Lopes, Milene C. Vallejo, Alisson L. Matsuo, Igor C. Almeida, Rosana Puccia

**Affiliations:** 1 Departamento de Microbiologia, Imunologia e Parasitologia, Escola Paulista de Medicina-Universidade Federal de São Paulo, EPM-UNIFESP, São Paulo, Brazil; 2 Border Biomedical Research Center, Dept. of Biological Sciences, University of Texas at El Paso (UTEP), El Paso, Texas, United States of America; Instituto de Salud Carlos III, Spain

## Abstract

**Background:**

The fungal cell wall is a complex and dynamic outer structure. In pathogenic fungi its components interact with the host, determining the infection fate. The present work aimed to characterize cell wall lipids from *P. brasiliensis* grown in the presence and absence of human plasma. We compared the results from isolates Pb3 and Pb18, which represent different phylogenetic species that evoke distinct patterns of experimental paracoccidioidomycosis.

**Methodology/Principal Findings:**

We comparatively characterized cell wall phospholipids, fatty acids, sterols, and neutral glycolipids by using both electrospray ionization- and gas chromatography-mass spectrometry analyses of lipids extracted with organic solvents followed by fractionation in silica-gel-60. We detected 49 phospholipid species in Pb3 and 38 in Pb18, including phosphatidylcholine, phosphatidylethanolamine, phosphatidylserine, phosphatidylglycerol, phosphatidylinositol, and phosphatidic acid. In both Pb3 and Pb18, PC and PE had the most numerous species. Among the fatty acids, C18∶1 and C18∶2 were the most abundant species in both isolates, although C18∶2 was more abundant in Pb18. There was a different effect of plasma supplementation on fatty acids depending on the fungal isolate. The prevalent glycolipid species was Hex-C18∶0-OH/d19∶2-Cer, although other four minor species were also detected. The most abundant sterol in all samples was brassicasterol. Distinct profiles of cell wall and total yeast sterols suggested that the preparations were enriched for cell wall components. The presence of plasma in the culture medium specially increased cell wall brassicasterol abundance and also other lipids.

**Conclusions/Significance:**

We here report an original comparative lipidomic analysis of *P. brasiliensis* cell wall. Our results open doors to understanding the role of cell wall lipids in fungal biology, and interaction with anti-fungal drugs and the host.

## Introduction

The cell wall is a complex and dynamic fungal structure involved in functions such as maintenance of the cell shape, protection from mechanical injuries, adaptation to morphogenesis, and withstanding osmotic pressure [1]. It is the main structure in contact with the host, therefore directly participating in host-fungal relationship [2]. Its main constituents are absent in mammalian cells, which makes them potential pharmacological targets [3]. The main constituents of fungal cell wall are structural polysaccharides, specifically glucans and chitin, but an outer layer of mannan and several glycoproteins have also been described [3].

Dimorphic fungi constitute a small group of pathogens that develop as yeast or mycelia according to the temperature of growth [4]. In this group, the cell wall lipid content varies with the fungal species and/or morphological phase, ranging from 5.5%, in *Blastomyces dermatitidis* pathogenic yeasts, to 26% in *Sporothrix schenckii* environmental hyphae [5]. The role of lipids in the fungal cell wall has not been fully elucidated. The best characterized lipid species in opportunistic *Candida albicans* cell wall is phospholipomannan, which takes part in fungal resistance to cell wall-disturbing agents and pathogenicity [6]. Glucosylceramide (GlcCer) species have already been identified in opportunistic *Cryptococcus neoformans* cell wall, being implicated in budding, growth, and pathogenicity [7], [8].

Our fungal model is thermodimorphic fungus *Paracoccidioides brasiliensis* that causes paracoccidioidomycosis (PCM), a prevalent systemic granulomatous mycosis in Latin America [9]. *P. brasiliensis* develops as hyphae in the environment and as multibudding yeasts in the host. Infection starts with inhalation of environmental conidia, whereas the outcome of an active disease and severity of clinical forms will depend on the strain virulence and the type of immune response that it evokes [10]. Kanetsuna and coworkers [11] studied the chemical composition of carefully isolated cell wall preparations from *P. brasiliensis* yeasts and mycelia and found a proportion varying from 5 to 11% lipids in both morphological forms. In yeast mutants, lipids concentrated in a basic/acid soluble cell wall fraction, representing up to 80% of its contents [12], [13]. The nature of *P. brasiliensis* cell wall lipids, however, has been poorly studied, although some possibly surface glycosphingolipid species [14] have been characterized and shown to be important for fungal growth and differentiation [15].

The aim of the present work was to use both electrospray ionization- (ESI-) and gas chromatography-mass spectrometry (GC-MS) analyses to characterize yeast-form cell wall lipids from two phylogenetically distinct *P. brasiliensis* Pb3 and Pb18 isolates [16]. We also investigated the effects caused by human plasma on the cell wall lipid composition. We were able to comparatively characterize phospholipids, fatty acids, sterols, and neutral glycolipids.

## Materials and Methods

### Reagents

Otherwise indicated, all reagents used in this study were of molecular biology-, HPLC-, or mass spectrometry-grade from Sigma-Aldrich (St. Louis, MO).

### *P. brasiliensis* and Growth Conditions

Isolates Pb3 and Pb18 from *P. brasiliensis* (described in [17]) were maintained at 36°C in solid, modified YPD medium (0.5% yeast extract, 0.5% casein peptone, 1.5% glucose, pH 6.5). For cell wall isolation, yeast cells were transferred from 7-day-old solid, modified YPD into 200 mL of Ham’s F12 medium (Invitrogen) complemented with 1.5% glucose (F12/Glc) and supplemented or not with 2% heat-inactivated (56°C, one hour) human plasma. The human plasma used to supplement fungal cultures in the study was supplied by the blood bank from Hospital São Paulo (Hemocentro do Hospital São Paulo). It was from a pool of plasma samples with no identifiers that allow samples to be linked to individually identifiable living human subjects or associated medical information. Therefore, this study is considered exempt of human subjects protocol, strictly following the guidelines for human subjects of the Comitê de Ética em Pesquisa da Universidade Federal de São Paulo/Hospital São Paulo and the National Institutes of Health (UNIFESP Ethics Committee, approval protocol number 0366/07). Pre-inoculums were cultivated under shaking at 36°C for 4 days and the yeast cells collected from four 200-mL flasks were transferred to fresh medium (500 mL) and cultivated for 2 days for cell wall purification. Cells were analyzed for viability (>95%) with trypan blue. It is worth mentioning that before using the plasma-supplemented media protein precipitation was observed during sterilization tests at 36°C. Precipitates were further eliminated with a second filtration round; therefore, the final plasma concentration is unknown.

### Cell Wall Purification

Yeasts were washed 3 times with phosphate-buffered saline solution (PBS) and mechanically disrupted with glass beads (425–600 µm, Sigma Aldrich) in a cell disruptor (B. Braun Biotech International GmBH, Melsungen, Germany) (6 times for 10 min, alternating with 10 min on ice). Yeast cell debris containing cell wall were pelleted by centrifugation (5,000×*g* for 10 min at 4°C) and washed with deionized water. Cell wall was separated from cytoplasmic and membranous contents by three sequential centrifugations (8,000×*g* for 45 min at 4°C) in 85% sucrose [11]. Cell wall fractions were then washed 50 times with ultrapure, deionized water (Milli-Q, Millipore), and lyophilized. Previato et al. [18] established this cell wall washing protocol for *Sporothrix schenckii*, and showed by transmition electron microscopy that plasma membrane was absent from final preparations.

### Lipid Extraction and Fractionation

Yeasts and a pool of numerous cell wall preparations (100 mg) from Pb3 and Pb18 were sequentially extracted three times with one mL chloroform:methanol (2∶1, v:v) and chloroform:methanol:water (2∶1∶0.8, v:v:v) under shaking with glass beads, as described in Yichoy et al. [19]. Lipids from both extractions were mixed and dried under N_2_ stream. Lipid fractionation was performed in a Pasteur pipette containing 500 mg silica-gel-60 spheres (60 Å, 200–400 mm, Sigma-Aldrich, St. Louis, MO). The column was washed sequentially with 5 mL each HPLC-grade methanol, acetone, and chloroform, and lipids dissolved in 2 mL chloroform were loaded onto it. Sterols were eluted with 5 mL chloroform, followed by neutral glycolipids elution with 5 mL acetone and phospholipids with 5 mL methanol. Lipid fractions were collected and dried under N_2_ stream. Lipid data is due to one experimental set for each isolate grown in two parallel growth conditions (with and without human plasma).

### Glycolipids Permethylation

Glycolipids were dried under N_2_ stream and stored overnight at −20°C with desiccant silica, resuspended in 150 µL anhydrous dimethyl sulfoxide (DMSO), vortexed with few milligrams of powdered, anhydrous NaOH, mixed with 80 µL iodomethane, and incubated under shaking for 1 hour at room temperature. Deionized water (2 mL) and dichloromethane (2 mL) were then added and the mixture was vortexed and centrifuged to remove the aqueous upper layer. The organic phase was washed three times with 2 mL deionized water and dried under N_2_ stream.

### Fatty Acids Methylation

Fatty acids methylation was performed as described elsewhere [20]. Phospholipids were incubated in 400 µL 13 M ammonium hydroxide (NH_4_OH):methanol (1∶1, v:v) for 1 h at 37°C, dried under N_2_ stream, and washed twice with anhydrous methanol. Samples were incubated in 400 µL 0.5 M methanolic HCl (Supelco, Sigma-Aldrich) for 1 h at 75°C and neutralized with 400 µL 0.5 M NaCl. Samples were then partitioned with 1.5 mL deionized water and 1.5 mL dichloromethane, and the fatty acid-containing organic phase was washed twice in deionized water and concentrated to 100 µL under N_2_ stream.

### ESI-MS/MS Analysis of Phospholipids and Glycolipids

Phospholipids and glycolipids were analyzed by electrospray ionization tandem mass spectrometry (ESI-MS/MS) on a linear ion-trap mass spectrometer (LTQ XL, ThermoFisher Scientific, San Jose, CA) coupled with an automated nanospray source (Triversa NanoMate System, Advion). Phospholipids were analyzed in negative- and positive-ion modes. For the negative-ion mode analysis, samples were redissolved in methanol containing 0.05% formic acid (FA) and 0.05% NH_4_OH; 2.5 µM phosphatidylglycerol (C12∶0/C12∶0-PG) (Avanti Polar Lipids, Alabaster, AL) was used as internal standard. In the positive-ion mode, samples were redissolved in 10 mM LiOH/methanol and 2.5 µM phosphatidylcholine (C11∶0/C11∶0-PC) (Avanti Polar) was used as internal standard. Full-scan spectra were collected at the 500–1000 *m/z* range, and samples were subjected to total-ion mapping (TIM) (2 a.m.u. isolation width; pulsed-Q dissociation (PQD) to 29% normalized collision energy; activation Q of 0.7; and activation time of 0.1 ms). Permethylated glycolipids were dissolved in methanol and analyzed as described for phospholipids, with full-scan spectra acquisition at the 500–2000 *m/z* range and the following TIM settings: 700–900 *m/z* range, 2 a.m.u. isolation width; PQD to 32% normalized collision energy; activation Q of 0.7; and activation time of 0.1 ms. MS/MS spectra from both phospholipids and glycolipids were analyzed manually according to Pulfer and Murphy [21].

### GC-MS Analysis of Sterols

Sterols were redissolved in dichloromethane and analyzed by gas chromatography-mass spectrometry (GC-MS, Trace GC Polaris Q, Thermo Fisher Scientific) using a TR5-MS column (30 m x 250 µm×0.25 µm, Thermo Fisher Scientific). The injector temperature was maintained at 250°C. The column temperature was initially kept at 170°C for 3 min and then increased in a rate of 20°C/min up to 280°C and held at this temperature for 17 min. Helium was used as carrier gas at a flow rate of 1.2 mL/min. Ionization was performed in the positive-ion mode by electron impact at 70 eV and 200°C. The spectra were collected at the 50–650 *m/z* range and analyzed by searching the spectral library (NIST library, available at Xcalibur 1.4 Srl, Thermo Fisher Scientific) and by comparison to external standards of brassicasterol, ergosterol, and lanosterol (Avanti Polar Lipids), and cholesterol (Sigma-Aldrich). Relative quantification was performed by comparison with stigmasterol (Avanti Polar Lipids) internal standard.

### GC-MS Analysis of Fatty Acids

One microliter of methylated fatty acids in dichloromethane was used for GC-MS analysis. Samples were separated in a SP-2380 column (30 m x 250 µm×0.20 µm, Supelco). The injector was kept at 200°C, and the following temperature gradient was used: 70°C for 5 min, a 4°C/min increment up to 140°C, 2°C/min up to 185°C, and 185°C for 10 min. Helium was used as carrier gas at 1 mL/min. Ionization was performed in the positive-ion mode by electron impact with 70 eV, at 200°C. The spectra were collected at 30–400 *m/z* range, and extracted ion chromatograms were generated by plotting the diagnostic fragment-ion species at *m/z* 41, 43, and 55. Identification was carried out by fatty-acid species comparison to a FAME 37 mix external standard (Supelco).

## Results

*P. brasiliensis* lipids from carefully isolated cell wall preparations were extracted and fractionated in a silica-60 column into phospholipids, neutral glycolipids, and sterols. Total yeast cell lipids were analyzed for comparison. We compared the results for Pb3 and Pb18 cultivated in the presence or absence of human plasma. These isolates were chosen because they represent distinct phylogenetic species [16], which evoke infection with different profiles in B10.A mice [22].

### ESI-MS/MS Analysis of Cell Wall Phospholipids

**Figure 1:**
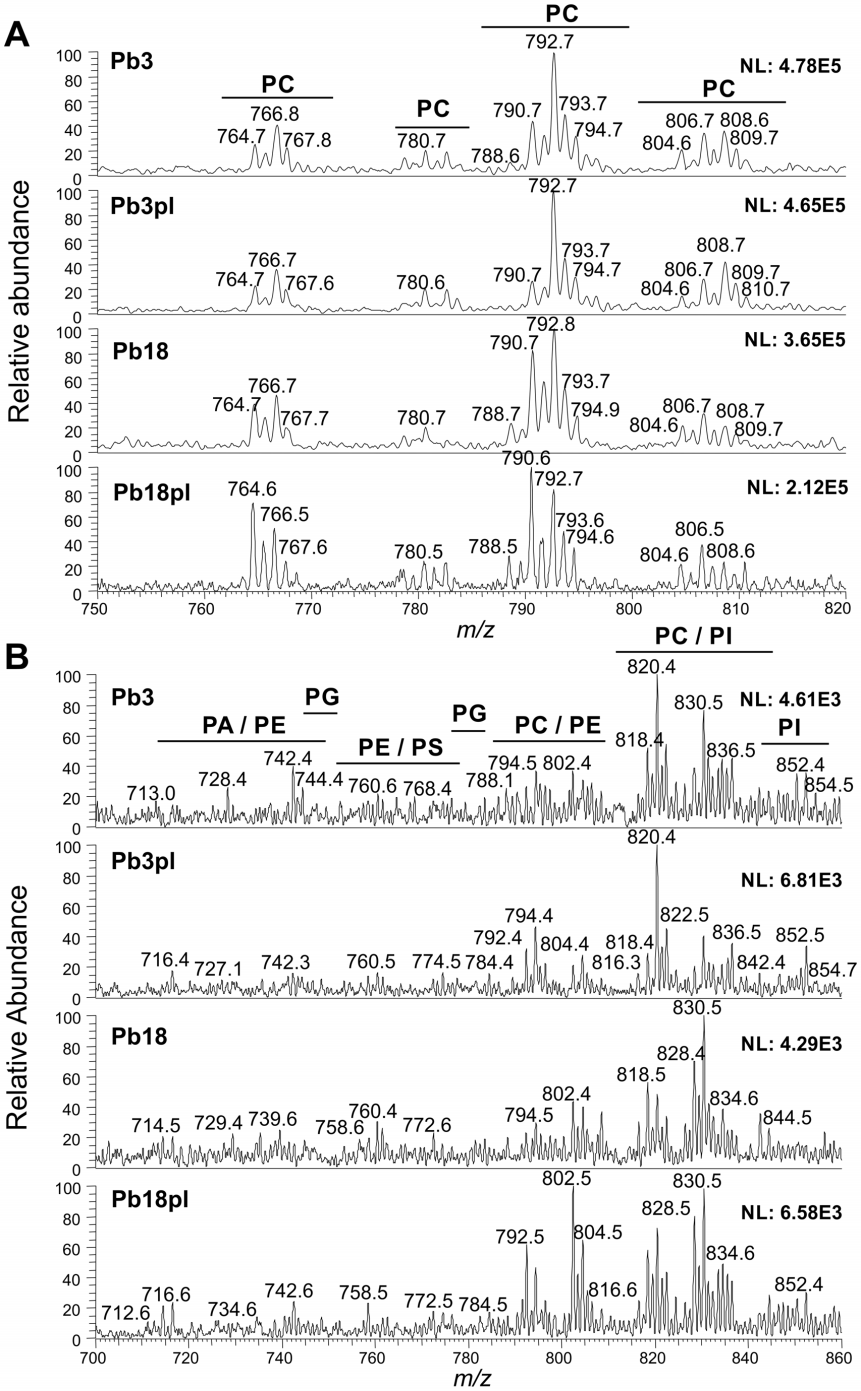
Full-scan spectra of cell wall phospholipids from Pb3 and Pb18 cultivated in the presence (pl) or absence of plasma. ESI-MS analysis in (A) positive- and (B) negative-ion modes are shown. *m/z*, mass to charge ratio. Assigned peaks are indicated.

Phospholipids from Pb3 and Pb18 cell wall preparations were ionized in positive- (Fig. 1A) and negative-ion (Fig. 1B) modes of ESI-MS/MS. Full-scan spectra showed that the peak profiles were similar among isolates grown in the presence (pl) or absence of plasma, thus suggesting similar phospholipid composition. However, we found differences in phospholipid species abundance when comparing Pb18 and Pb3 grown in the absence of plasma, while a detailed study of species in the Pb18pl and Pb3pl samples was not carried out.

Phospholipid identification was performed by searching for diagnostic fragment ions. In the positive-ion mode, phosphatidylcholine (PC) species were found with an adduct of Na^+^ or Li^+^, or singly protonated (H^+^) following the 59-Da neutral loss, of trimethylamine ((CH_3_)_3_N or Me_3_N). Fatty acid chains were identified by neutral-loss fragments ([M – fatty acid+Li^+^ (or Na^+^)]^+^) or loss of both fatty acids and Me_3_N ([M – fatty acid – Me_3_+ Li^+^ (or Na^+^)]^+^), as exemplified in MS/MS spectrum of the major PC species (C18∶1/C18∶1-PC) (Fig. S1). In the negative-ion mode, diagnostic fragment ions at *m/z* 171, corresponding to glycerophosphate (GroP – H)^-^, and at *m/z* 153, corresponding to dehydrated glycerophosphate (GroP – H_2_O – H)^-^ were used to all phospholipids, which were then identified with chloride (Cl^-^) or formate (HCOO^-^) adducts. For instance, neutral losses of 50 or 60 Da, related to the N-methyl group associated to a Cl^-^ [M – MeCl – H]^-^ or a formate [M – HCOOMe – H]^-^, respectively, were used in PC identification (Fig. S2). In addition, specific diagnostic ions were used to identify each phospholipid class. Phosphatidylethanolamine species (PE) were identified by the *m/z* 196 ion, corresponding to dehydrated glycerophosphoethanolamine [GroPEtN – H_2_O – H]^-^ (Fig. S3). The 87-Da neutral loss of the serine headgroup [M – Ser – H]^-^ and the diagnostic ion *m/z* 241, corresponding to the dehydrated phosphoinositol [InsP – H_2_O – H]^-^, were used in the identification of phosphatidylserine (PS) (Fig. S4) and phosphatidylinositol (PI) species, respectively (Fig. S5). Since there are no specific neutral losses or diagnostic ions characteristic of phosphatidylglycerol (PG) (Fig. S6) and phosphatidic acid (PA) (Fig. S7), their identification was achieved by considering the molecular mass of intact parent ions. Fatty acids were identified by the *m/z* ratio of the negatively charged carboxylate ions at *m/z* 255 (C16∶0), 279 (C18∶2), and 281 (C18∶1) (Figs. S6 and S7).

**Figure 2:**
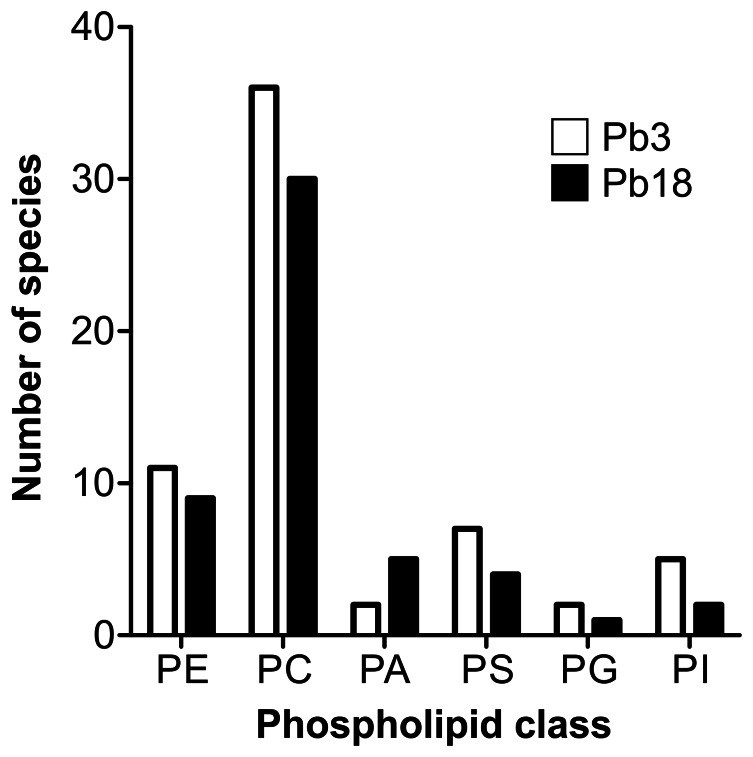
Number of phospholipid species from each class identified in Pb3 and Pb18 cell wall cultivated in the absence of plasma. ESI-MS/MS analysis in both negative- and positive-ion modes. Detailed description of individual phospholipid species is found in Table 1.

Detected phospholipids showed *m/z* ions between *m/z* 556.5 and 851.7 in the negative-ion mode, and between *m/z* 526.5 and 822.7 in the positive-ion mode. Phospholipids of PC, PE, PG, PI, PS, and PA classes were identified in both Pb3 and Pb18 (Table 1, Fig. 2). In total, we identified more phospholipid species in Pb3 (63) than in Pb18 (51), as shown in Table 1 and Fig. 2. In both Pb3 and Pb18, PC and PE classes had the most numerous species, followed by PS, PA/PI, and PG. However, Pb18 showed more PA (5) than PS (4) species, while PI (2) and PG (1) were underrepresented. In both isolates there were *lyso*-C18∶1-PC and *lyso*-C18∶2-PC, but an C18∶1-alkyl/C18∶1-acyl-PE species (plasmalogen or plasmenylethanolamine) was only detected in Pb3 (Table 1).

**Table 1:** Composition and relative abundance of major phospholipids from Pb3 and Pb18 cell wall identified by ESI-MS total-ion mapping.

Ion mode	Observed species					Proposed composition	Predicted	Normalized	
							mass (Da)	intensity^a^	
	Pb3			Pb18					
	Ion species	*m/z*		Ion species	*m/z*			Pb3	Pb18
**Phosphatidylethanolamine (PE)**									
(−)	[M − H]^−^	714.6		[M − H]^−^	714.6	C16∶0/C18∶2	715.5	TR^c^	0.5
(−)	[M − H]^−^	728.7		[M − H]^−^	728.7	C16∶0/C19∶2	729.5	0.2	0.3
(−)	[M − H]^−^	742.7		[M − H]^−^	742.7	C18∶1/C18∶1	743.5	0.3	0.3
(−)	[M − H]^−^	746.6		ND	ND	C16∶0/C20∶0	747.6	0.4	ND
(−)	[M − H]^−^	756.6		[M − H]^−^	756.7	C18∶1/C19∶1	757.5	0.4	0.4
(−)	[M − H]^−^	770.6		ND	ND	C18∶1/C20∶1	771.5	0.6	ND
(−)	ND	ND		[M − H]^−^	716.7	C16∶0/C18∶1	717.5	ND	0.5
(−)	[M − H]^−^	786.6		ND	ND	C18∶1/C21∶0	787.5	0.5	ND
(−)	[M+Cl]^−^	808.7		ND	ND	C18∶1/C20∶0	773.5	0.4	ND
**Phosphatidylcholine (PC)**									
(+)	[M+Li]^+^		528.4	[M+Li]^+^	528.5	*lyso*−C18∶1	522.3	0.4	0.2
(−)	[M+HCOO]^−^		748.7	ND	ND	C14∶1/C16∶0	704.6	0.3	ND
(−)	[M+HCOO]^−^		776.6	ND	ND	C16∶0/C16∶1	732.6	0.5	ND
(−)	[M+Cl]^−^		792.7	[M+Cl]^−^	792.6	C16∶0/C18∶2	758.6	1.6	0.5
(+)	[M+Li]^+^		764.6	[M+Li]^+^	764.6			1.5	1.4
(−)	[M+HCOO]^−^		804.6	[M+HCOO]^−^	804.7	C16∶0/C18∶1	760.6	2.1	1.6
(+)	[M+Li]^+^		766.7	[M+Li]^+^	766.6			2.0	1.4
(+)	[M+Na]^+^		778.7	ND	ND	C16∶0/C18∶3	756.6	0.5	ND
(−)	[M+Cl]^−^		808.7	[M+HCOO]^−^	818.7	C16∶0/C19∶1	774.6	0.6	1.0
(−)	[M+Cl]^−^		816.6	ND	ND	C18∶2/C18∶2	782.6	1.2	ND
(+)	[M+Li]^+^		788.7	[M+Li]^+^	788.9			1.0	1.0
(−)	ND		ND	[M+HCOO]^−^	842.7	C18∶2/C19∶1	798.6	ND	0.8
(−)	[M+Cl]^−^		818.6	[M+Cl]^−^	818.7	C18∶1/C18∶2	784.6	0.6	1.0
(+)	[M+Li]^+^		790.6	[M+Li]^+^	790.6			2.5	2.7
(−)	[M+Cl]^−^		821.6	[M+Cl]^−^	820.7	C18∶1/C18∶1	786.6	2.3	3.2
(+)	[M+Li]^+^		792.6	[M+Li]^+^	792.6			4.3	3.0
(−)	[M+Cl]^−^		822.5	ND	ND	C16∶0/C20∶1	788.6	1.2	ND
(+)	[M+Li]^+^		796.7	[M+Li]^+^	796.6	C18∶0/C18∶0	790.6	0.5	0.2
(−)	[M+Cl]^−^		822.5	ND	ND	C18∶0/C18∶1	788.6	1.2	ND
(+)	[M+Li]^+^		794.6	[M+Li]^+^	794.6			1.5	0.9
(−)	[M+HCOO]^−^		844.7	[M+HCOO]^−^	844.7	C18∶1/C19∶1	800.6	0.8	0.4
(+)	[M+Na]^+^		822.6	[M+Na]^+^	822.7			0.7	0.3
(−)	[M+Cl]^−^		850.6	ND	ND	C18∶1/C20∶0	816.6	1.0	ND
(+)	[M+Li]^+^		818.6	[M+Li]^+^	818.6	C19∶1/C19∶2	812.6	0.3	0.3
**Phosphatidic Acid (PA)**									
(−)	[M − H]^−^		673.5	[M − H]^−^	673.6	C16∶0/C18∶1	674.5	TR	0.4
(−)	[M − H]^−^		699.6	[M − H]^−^	699.6	C18∶1/C18∶1	701.0	0.3	0.2
(−)	ND		ND	[M − H]^−^	687.6	C16∶0/C19∶1	688.5	ND	0.3
**Phosphatidylserine (PS)**									
(−)	[M − H]^−^		758.5	[M − H]^−^	758.7	C16∶0/C18∶2	759.5	0.3	0.4
(−)	[M − H]^−^		760.6	[M − H]^−^	760.7	C16∶0/C18∶1	761.5	0.4	0.3
(−)	[M − H]^−^		774.6	[M − H]^−^	774.6	C16∶0/C19∶1	775.5	0.3	0.3
(−)	[M − H]^−^		786.6	ND	ND	C16∶0/C20∶2	787.5	0.3	ND
(−)	[M − H]^−^		786.6	ND	ND	C18∶1/C18∶1	787.5	0.3	ND
(−)	[M − H]^−^		790.7	ND	ND	C16∶0/C20∶0	791.5	0.4	ND
**Phosphatidylglycerol (PG)**									
(−)	[M − H]^−^		747.7	[M − H]^−^	747.7	C16∶0/C18∶1	748.5	0.4	0.2
**Phosphatidylinositol (PI)**									
(−)	[M − H]^−^		833.7	[M − H]^−^	833.7	C16∶0/C18∶2	834.5	0.9	0.5
(−)	[M − H]^−^		835.7	[M − H]^−^	835.8	C16∶0/C18∶1	836.5	0.4	0.5
(−)	[M − H]^−^		847.6	ND	ND	C16∶0/C19∶2	848.5	0.4	ND

Only species with normalized intensity higher than 0.2 in at least one isolate is shown. ^a^ Normalized to the intensity of the internal standards C12∶0/C12∶0-PG (at *m/z* 609.5) and C11∶0/C11∶0-PC (at *m/z* 616.5) in the negative and positive–ion modes, respectively. ^b^ ND, not detected. ^c^ TR, trace amounts.

Abundance estimation (Table 1) was performed by normalizing each phospholipid species signal intensity to the internal standards C12∶0/C12∶0-PG (*m/z* 609.5) (in negative-ion mode) and C11∶0/C11∶0-PC (*m/z* 600.5) (in positive-ion mode). In general, the abundance of species between Pb3 and Pb18 were comparable, but some differences deserve to be highlighted. Two PC species, C16∶0/C20∶1-PC (*m/z* 822.5) and C18∶1/C20∶0-PC (*m/z* 850.6) were abundant in Pb3, but not in Pb18. On the other hand, C18∶2/C19∶1-PC (*m/z* 842.7) and C16∶0/C18∶2-PA (*m/z* 739.6) were not detected in Pb3 but were abundant in Pb18 cell wall.

### Compositional Analysis of Cell Wall Phospholipid Fatty Acids by GC-MS

Fatty acids of cell wall and total yeast cell phospholipids from Pb3 and Pb18 cultivated in the presence (pl) and absence of plasma were methylated and analyzed by GC-MS. Fatty acids were identified by comparison with an external standard mix and were shown to have double bonds in *cis* configuration. Although we have not been able to determine the unsaturation position, we assumed it happened in the most common positions, specifically, between C9 and C10 (Δ^9^), in monounsaturated fatty acids, or in both Δ^12^ and Δ^15^ [23]. Relative abundance of fatty acids was estimated by normalization to the C16∶0 peak area.

**Figure 3:**
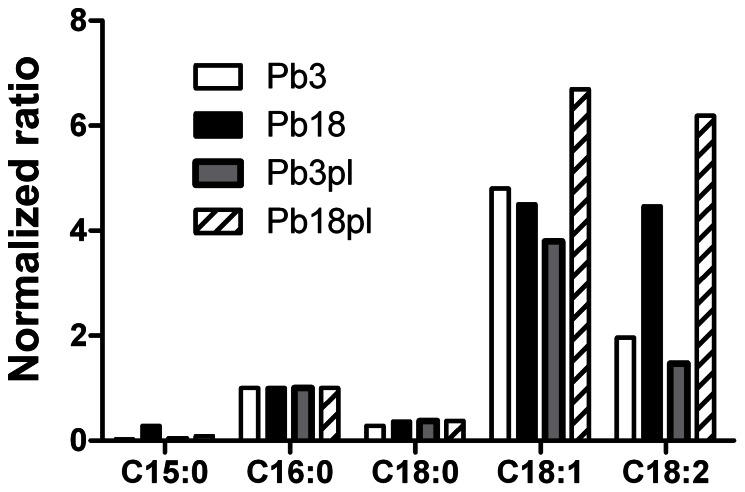
GC-MS analysis of cell wall phospholipid fatty acids from Pb3 and Pb18 cultivated in the presence (pl) or absence of plasma. Fatty acid abundance was normalized to the C16∶0 peak area.

Cell wall fatty acid composition was similar in all samples analyzed (Fig. 3): oleic acid (C18∶1) and linoleic acid (C18∶2) prevailed, whereas palmitic acid (C16∶0), stearic acid (C18∶0), and pentadecanoic acid (C15∶0) were minor components. However, noticeable differences in abundance occurred, especially when the isolates were growing in the presence of plasma. Although C18∶1 was equally abundant in both isolates, C18∶2 predominated in Pb18. Moreover, the presence of plasma induced in Pb18 an increase in both fatty acid abundances, while they slightly decreased in Pb3.

Taken together, fatty acid analysis indicated abundance differences in cell wall fatty acid composition between Pb3 and Pb18, including a different effect of plasma depending on the fungal isolate.

### GC-MS Analysis of Cell Wall Sterols

Sterol fractions eluted from a silica-60 column with chloroform were analyzed by GC-MS and the species were identified by comparing with external standards or searching a reference spectral library. Relative abundance analysis was accomplished by normalization to stigmasterol (internal standard) peak area. Total yeast cell sterols were also analyzed for comparison.

**Figure 4:**
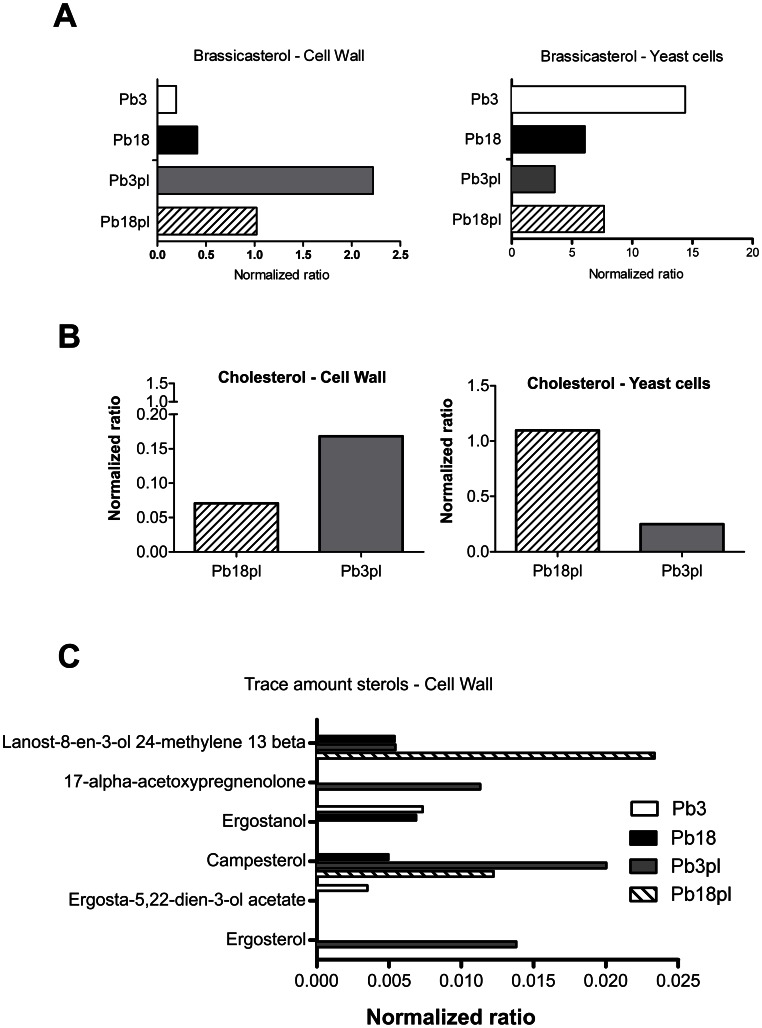
Comparison of cell wall and total yeast cell sterols from Pb3 and Pb18 cultivated in the presence (pl) or absence of plasma. Sterols were analyzed by GC-MS and abundance was normalized to the stigmasterol (internal standard). A, Comparison of brassicasterol amounts, the most the most abundant sterol detected. B, Cholesterol uptake from plasma by Pb3 and Pb18 isolates. C, Comparison of sterols found in trace amounts in cell wall samples.

Brassicasterol (ergosta-5,22-dien-3β-ol) was the most abundant sterol identified in Pb3 and Pb18 cell wall and also within total lipids (Fig. 4A). In yeast cells growing in the absence of plasma, brassicasterol was relatively more abundant in Pb18 than Pb3 cell wall preparations. However, in total lipids from whole cell preparations the proportion was about 4-fold higher in Pb3. Plasma modified this scenario more drastically in cell wall brassicasterol, whose abundance increased in both Pb18 and Pb3, although in Pb3 it showed a more pronounced effect (11-fold against 2-fold in Pb18) (Fig. 4A). Total Pb3 brassicasterol, on the contrary, decreased in the presence of plasma, whereas in Pb18 it slightly increased (Fig. 4A).

Cholesterol was identified only in samples from *P. brasiliensis* cultivated in the presence of plasma (Fig. 4B), indicating that it has been incorporated from the medium during fungal growth. Twice more cholesterol was incorporated onto Pb3pl than Pb18pl cell wall, but total cholesterol was 4-fold higher in Pb18pl.

Ergosterol, ergosta-5,22-dien-3-ol acetate, campesterol, ergostanol, 17-alpha-acetoxypregnenolone, and lanost-8-en-3-ol 24-methylene 13-beta were also detected in small amounts, which varied with isolate and growth condition (Fig. 4C). Intriguingly, some trace sterols seem to be considerably induced by plasma, especially in Pb3 (Fig. 4C).

### ESI-MS/MS Analysis of Cell Wall Glycolipids

**Figure 5:**
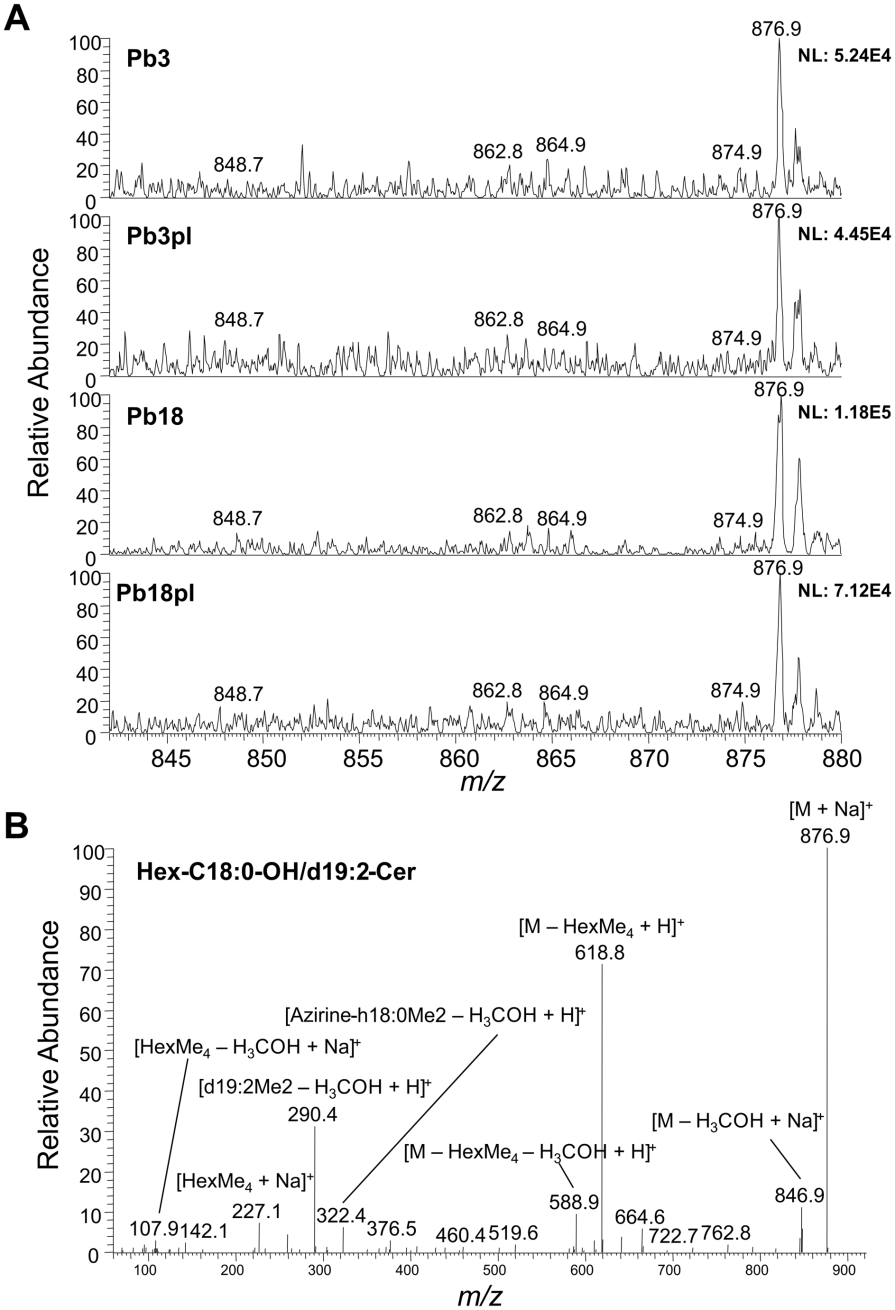
ESI-MS analysis of cell wall neutral glycolipids from Pb3 and Pb18 cultivated in the presence (pl) or absence of plasma. A, Full-scan spectra in the positive-ion mode show the identified glycolipids Hex-C16∶0-OH/d19∶2-Cer (*m/z* 848.7), Hex-C18∶0-OH/d18∶2-Cer (*m/z* 862.8), Hex-C18∶0-OH/d18∶1-Cer (*m/z* 864.9), Hex-C18∶1-OH/d19∶2-Cer (*m/z* 874.9), and Hex-C18∶0-OH/d19∶2-Cer (*m/z* 876.9). B, Tandem-MS spectrum of the major glycolipid species identified (*m/z* 876.9). *m/z*, mass to charge ratio.

Neutral glycolipids from Pb3 and Pb18 cell wall were isolated in a silica-60 column, permethylated, and subjected to positive-ion mode ESI-MS/MS analysis. Figure 5A shows one major glycolipid species identified in full-scan spectra, but four other were detected only by MS/MS analysis. This is due to the fact that during the parent-ion selection in the mass spectrometer most of the background is eliminated, thus making MS/MS analysis more sensitive than full-MS scans. The most abundant species in all the samples was identified as Hex-C18∶0-OH/d19∶2-Cer (at *m/z* 876.9), according to tandem-MS fragment manual analysis (Fig. 5B). Diagnostic fragment ions for glycolipids corresponding to methylated hexose at *m/z* 227.1 [HexMe_4_– H_3_COH+Na]^+^ and 259.2 [HexMe_4_+ Na]^+^ were observed. Neutral losses of both fragments generated the corresponding fragment ions at *m/z* 588.8 [M – HexMe_4_– H_3_COH+Na]^+^ and *m/z* 618.8 [M – HexMe_4_+ Na]^+^. A permethylated d19∶2 sphingoid base fragment [d19∶2Me_2_– H_3_COH+H]^+^ and a C18∶0 fatty acid attached to part of the sphingoid base [azirine-h18∶0Me_2_– H_3_COH+H]^+^ could be identified by the *m/z* 290.4 and *m/z* 322.4 fragment ions, respectively. A similar analysis was performed to characterize minor glycolipids identified as Hex-C16∶0-OH/d19∶2-Cer (*m/z* 848.7), Hex-C18∶0-OH/d18∶2-Cer (*m/z* 862.8), Hex-C18∶0-OH/d18∶1-Cer (*m/z* 864.6) and Hex-C18∶1-OH/d19∶2-Cer (*m/z* 874.7) (Figs. S8, S9, S10, S11).

Although our data did not allow to elucidate the position of unsaturation or the methyl and hydroxyl groups in the sphingoid base, Toledo and coworkers [24] have fully characterized two glycosphingolipid species from Pb18 yeasts and hyphae by nuclear magnetic resonance (NMR) and found that the sphingoid base is probably methylated at C9 and the unsaturation is in the *cis* conformation at C4 and C8. Therefore, the main neutral glycolipid here identified is probably *N*-2′-hydroxyoctadecanoate (4*E*, 8*E*)-9-methyl-4,8-sphingadienine (*m/z* 876.8).

## Discussion

The results reported in this work show the lipid composition, including phospholipids, fatty acids, sterols, and neutral glycolipids, of cell wall preparations from *P. brasiliensis* yeast cells, comparing isolates Pb3 and Pb18. The effect of plasma in the lipid composition of the cell wall was assessed by studying preparations from yeasts cultivated in the presence or absence of human plasma. Distinct profiles of cell wall and total yeast sterols suggested that the preparations were enriched for cell wall components.

Pb3 and Pb18 isolates belong to distant phylogenetic *P. brasiliensis* groups and represent, respectively, the PS2 and S1 species [16]. They had distinct infection profiles in B10.A [22], BALB/C, and C57BL6 mice (Carvalho et al., unpublished), where Pb3 was less virulent and evoked more protective immune responses than Pb18. Considering that cell wall is the outer fungal structure and that interaction between the fungus and the host through cell wall components is essential to establish a host-pathogen relationship (review in [2]), we believe that the present characterization of cell wall lipids might at least partially help to understand, in the future, differences in pathogenesis between these two isolates.

A significant portion of cell wall lipids probably derives from membranous extracellular vesicles, which have recently been described with more details in *C. neoformans, Histoplasma capsulatum*, *Saccharomyces cerevisiae* [25], [26], [27], and also in *P. brasiliensis* by our group [28]. These vesicles transport a variety of components to the extracellular milieu (reviewed in [29]), and thus they need to cross the cell wall. In *C. neoformans*, they also traverse a thick polysaccharide capsule, as seen in transmission electron microscopy (TEM) images [25]. During this traffic, some vesicles might never reach the extracellular milieu, but simply remain in the cell wall (or capsule) and play still unknown roles. In *C. neoformans*, vesicles carry capsule polysaccharides thus participating in capsule synthesis [25]. We have recently described the lipid composition of extracellular vesicle preparations from Pb18 and Pb3 [30]. Comparison of the vesicle data and our present results points to more similarities than discrepancies, therefore suggesting that extracellular vesicles contribute with a significant part of cell wall lipids. Details of this comparison are included in the paragraphs below.

In our analysis, phospholipids of PC, PE, PG, PI, PS, and PA classes were identified in both isolates, although Pb3 had higher amounts of phospholipid species than Pb18. Phospholipid class distribution was generally similar between isolates, where PC, followed by PE, were the phospholipid classes with the most numerous species. This result aligns with those from Pb3 and Pb18 extracellular vesicles [30], and also from total yeast and mycelia phospholipid of distinct isolates [31], [32]. *Lyso*-C18∶1-PC and *lyso*-C18∶2-PC were observed in small amounts in both Pb3 and Pb18 cell wall, but an C18∶1-alkyl/C18∶1-acyl-PE species could be detected only in Pb3. In extracellular vesicles, two ether phospholipids (C16∶0-alkyl/C18∶2- or C18∶1-acyl-PI) were described only in Pb18 [30], but not on the cell wall. Ether phospholipids were hypothesized to play a role as reservoirs for key lipid mediators in inflammatory cells [33] and alkyl-acyl species of phosphatidylethanolamine have already been described in small amounts in Friend erythroleukaemia (FEL) cells [34], rat polymorphonuclear leucocytes [35], and in rat mast cell derived exosomes [36]. In the protozoan parasite *Trypanosoma cruzi*, glycosylphosphatidylinositol (GPI) anchors containing an alkylacyl moiety with an unsaturated fatty acid (C18∶1 or C18∶2) strongly induce proinflammatory cytokines [37].

Overall, cell wall fatty acid composition was similar between isolates; however, important differences in abundance can be highlighted. Similarly to extracellular vesicle fatty acids [30], oleic acid (C18∶1) cell wall amounts were comparable in Pb3 and Pb18, whereas linoleic acid (C18∶2) was more abundant in Pb18 than in Pb3. On the other hand, in a similar analysis recently performed by our group [30], total fatty acids identified in yeast cells were the same as in cell wall preparations, however with abundance differences: i) in Pb3 yeast C18∶2 was more abundant than C18∶1; ii) both C18∶1 and C18∶2 were more abundant in Pb3 than in Pb18, suggesting again that cell wall lipid preparations were enriched for this organelle. Previous data on fatty acids from various *P. brasiliensis* isolates described C16∶0 [31], C18∶1 [32], or C18∶2 [38] as the most abundant total fatty acids in yeast cells, suggesting that fatty acid composition can vary with the isolate and/or culture conditions.

In both Pb3 and Pb18 cell wall brassicasterol (ergosta-5,22-dien-3β-ol) was the prevalent sterol, and it was relatively more abundant in Pb18. That agrees with what we found in *P. brasiliensis* extracellular vesicles [30], and also in Pb3 and Pb18 total lipids (Fig. 4). The presence of brassicasterol in *P. brasiliensis* yeasts was originally described by San-Blas et al. [31], who also observed that in the mycelium phase ergosterol predominated instead. Lanosterol has already been described as an abundant sterol in *P. brasiliensis* yeasts [39], although in our analysis it has only been detected in trace amounts in the cell wall. Recently, brassicasterol and lanosterol were shown to be sterol precursors in *P. brasiliensis* [40].

We were able to identify five different species of neutral glycosphingolipids in cell wall preparations from both Pb3 and Pb18. In our analysis, only neutral glycolipids could be extracted, and whether acidic glycosphingolipid species are present on the cell wall still needs to be determined. The most abundant Hex-C18∶0-OH/d19∶2-Cer, and also Hex-C18∶1-OH/d19∶2-Cer, were identified in similar relative amounts in our previous analysis of extracellular vesicles [30], as well as in Pb18 yeast and hyphal cells [24]. The latter work identified glucose as the hexose by nuclear magnetic resonance (NMR). We consequently assume that the neutral glycolipids here identified are glucosylceramide (GlcCer) species, which have already been localized at *P. brasiliensis* yeast surface [14], besides *C. neoformans* [7] and *C. albicans* [41] cell wall and *C. neoformans* extracellular vesicles [25]. In *C. albicans,* the antifungal plant defensin RsAFP2 interacts with cell wall GlcCer causing cell wall stress and ceramide accumulation [41]. GlcCer was not recognized by paracoccidioidomycosis patients’ sera, although an acidic glycosphingolipid species (Pb-1) containing a terminal Gal*f* residue has been described as immunogenic [42]. GlcCer is a key element to *A. fumigatus* growth [43], *C. albicans* virulence [44], and *C. neoformans* budding, growth and pathogenicity [7], [8]. However, in *P. brasiliensis*, monoclonal antibodies directed to GlcCer were not able to promote a considerable inhibition of fungal differentiation and colony formation [15]. Therefore, the role of GlcCer in the cell wall remains unknown.

In the present work we observed fluctuations in lipid composition when *P. brasiliensis* was cultivated in human plasma. In general, many lipids were detected in proportionally higher amounts when yeasts were cultivated in plasma-containing medium. That was also observed by Chattaway and coworkers [44] in cell wall lipids when *C. albicans* hyphae and yeasts were cultivated in the presence of serum. Moreover, the influence of human plasma in the transcriptional profile of *P. lutzii* (Pb01) yeast cells showed upregulated transcription of genes related to fatty acid degradation and cell wall remodeling [45]. Plasma-containing medium induced an increase in both C18∶1 and C18∶2 amounts in Pb18, whereas it evoked the opposite effect in Pb3. It has already been described that culture temperature, age and medium composition are able to change fungal fatty acid distribution [46], [47], [48], which reinforces our findings. The most dramatic effect of plasma was observed in Pb3 cell wall brassicasterol that increased 11-fold in the presence of plasma, while yeast total brassicasterol rate decreased. We observed that the effect was isolate-dependent, since plasma did not change total Pb18 brassicasterol of yeast cells, although it evoked a two-fold increase in cell wall brassicasterol. Cholesterol incorporation rates in the cell wall were also distinct between isolates, being higher in Pb3, although total cholesterol rate was higher in Pb18. A possible interpretation to these results is that Pb18 yeast cells present a higher cholesterol intake in comparison to Pb3.

In brief, we compared the cell wall phospholipids, fatty acids, sterols, and neutral glycolipids from *P. brasiliensis* Pb3 and Pb18 and observed the effect of human plasma in composition and abundance. Despite some discrepancies, our data were similar to the lipid composition described by our group for Pb3 and Pb18 extracellular vesicles [30]. That supports the idea that extracellular vesicles are at least partially responsible for cell wall lipid composition. The present work substantially revealed the nature of *P. brasiliensis* cell wall lipid structure, thus opening doors to understanding its role in fungal biology, interaction with anti-fungal drugs, and with the host.

## Supporting Information

Figure S1 **Tandem-MS spectrum of C18∶1/C18∶1-PC (Li^+^ adduct), the most abundant PC species identified in the positive-ion mode.** Fragmentation was performed by total-ion mapping (TIM) using PQD and spectra were analyzed manually. ChoP, phosphatidylcholine; Me_3_N, trimethylamine. Assigned peaks are indicated. *m/z*, mass to charge ratio.
(PPTX)

Figure S2 **Tandem-MS spectrum of C18∶1/C18∶1-PC (HCOO^-^ adduct), the most abundant PC species identified in the negative-ion mode.** Fragmentation was performed by TIM using PQD and spectra were analyzed manually. GroP, glycerophosphate; Me, methyl. Assigned peaks are indicated.
(PPTX)

Figure S3 **Tandem-MS spectrum of C16∶0/C18∶1-PE, the most abundant PE species identified in the negative-ion mode.** Fragmentation was performed by TIM using PQD and spectra were analyzed manually. GroP, glycerophosphate. Assigned peaks are indicated.
(PPTX)

Figure S4 **Tandem-MS spectrum of C16∶0/C18∶1-PS, the most abundant PE species identified in the negative-ion mode.** Fragmentation was performed by TIM using PQD and spectra were analyzed manually. GroP, glycerophosphate; Ser, serine. Assigned peaks are indicated.
(PPTX)

Figure S5 **Tandem-MS spectrum of C16∶0/C18∶2-PI, the most abundant PI species identified in the negative-ion mode.** Fragmentation was performed by TIM using PQD and spectra were analyzed manually. GroP, glycerophosphate; Ins, inositol; InsP, phosphoinositol. Assigned peaks are indicated.
(PPTX)

Figure S6 **Tandem-MS spectrum of C16∶0/C18∶1-PG, the most abundant PG species identified in the negative-ion mode.** Fragmentation was performed by TIM using PQD and spectra were analyzed manually. GroP, glycerophosphate. Assigned peaks are indicated.
(PPTX)

Figure S7 **Tandem-MS spectrum of 16∶0/18∶2-PA, the most abundant PI acid species identified in the negative-ion mode.** Fragmentation was performed by total-ion mapping using PQD and spectra were analyzed manually. GroP, glycerophosphate. Assigned peaks are indicated.
(PPTX)

Figure S8 **Tandem-MS spectrum of the glycolipid Hex-C16∶0-OH/d19∶2-Cer identified at** ***m/z*** **848.7.** Fragmentation was performed in the positive-ion mode by TIM using PQD and spectra were analyzed manually. Assigned peaks are indicated.
(PPTX)

Figure S9 **Tandem-MS spectrum of the glycolipid Hex-C18∶0-OH/d18∶2-Cer identified at** ***m/z*** **862.8.** Fragmentation was performed in the positive-ion mode by total-ion mapping using pulsed-Q dissociation (PQD) and spectra were analyzed manually. Assigned peaks are indicated.
(PPTX)

Figure S10 **Tandem-MS spectrum of the glycolipid Hex-C18∶0-OH/d18∶1-Cer identified at** ***m/z*** **864.9.** Fragmentation was performed in the positive-ion mode by TIM using PQD and spectra were analyzed manually. Assigned peaks are indicated.
(PPTX)

Figure S11 **Tandem-MS spectrum of the glycolipid Hex-C18∶1-OH/d19∶2-Cer identified at** ***m/z*** **874.9.** Fragmentation was performed in the positive-ion mode by total-ion mapping using PQD and spectra were analyzed manually. Assigned peaks are indicated.
(PPTX)
