# Supplementary material for: Characterization of Cell Wall Lipids from the Pathogenic Phase of Paracoccidioides brasiliensis Cultivated in the Presence or Absence of Human Plasma
Source: PLoS One. 2013 May 17;8(5):e63372. doi: 10.1371/journal.pone.0063372 (PMC3656940; doi:10.1371/journal.pone.0063372)
Supplement: Figure S1 — Tandem-MS spectrum of C18∶1/C18∶1-PC (Li+ adduct), the most abundant PC species identified in the positive-ion mode. Fragmentation was performed by total-ion mapping (TIM) using PQD and spectra were analyzed manually. ChoP, phosphatidylcholine; Me3N, trimethylamine. Assigned peaks are indicated. m/z, mass to charge ratio. (PPTX) [file pone.0063372.s001.pptx]

## Slide 1
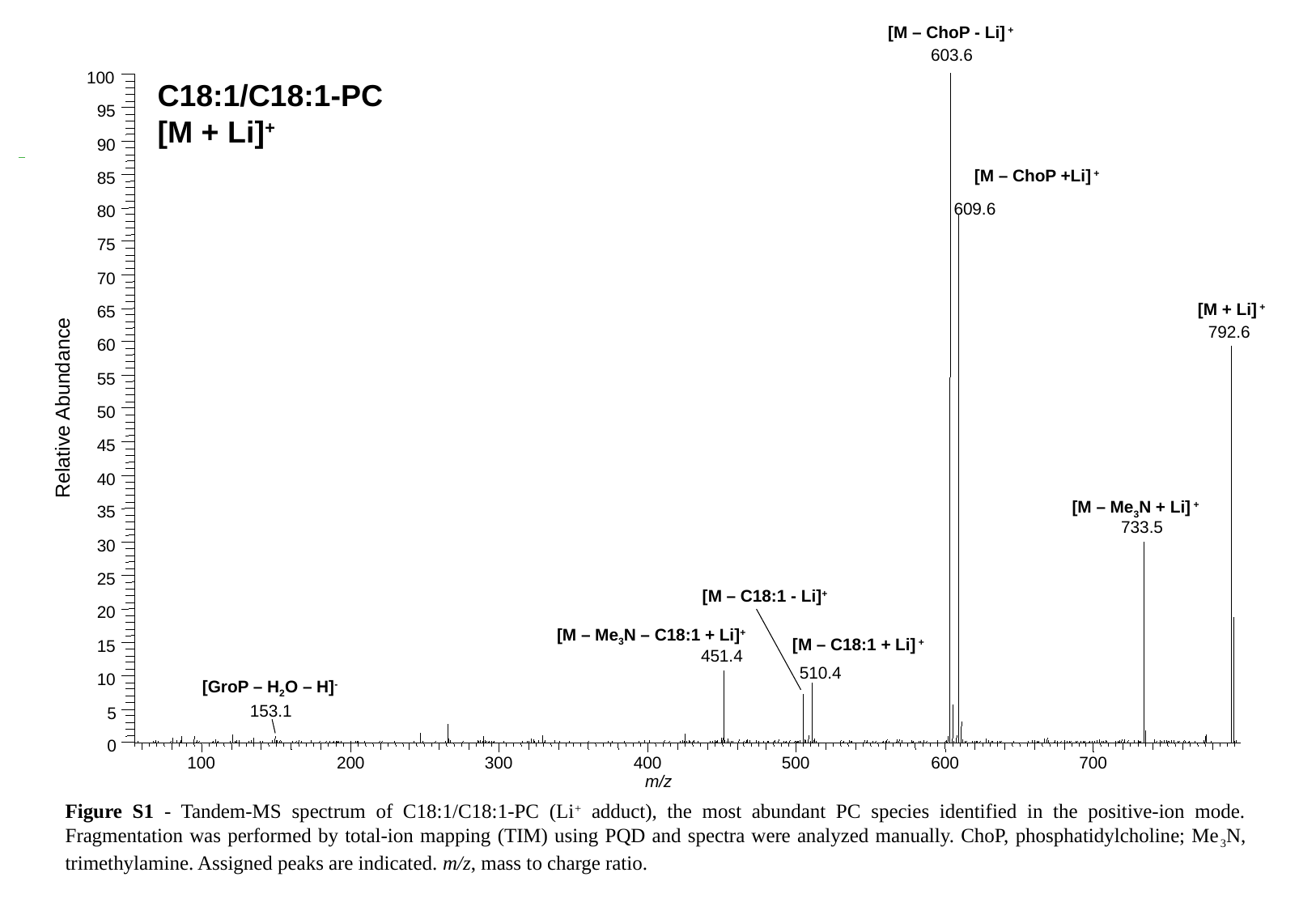

[M – ChoP - Li] +
100
200
300
400
500
600
700
m/z
603.6
100
95
90
85
80
75
70
65
60
55
Relative Abundance
50
45
40
35
30
25
20
15
10
5
0
609.6
792.6
733.5
451.4
510.4
[M – ChoP +Li] +
[M + Li] +
[M – Me3N + Li] +
[M – C18:1 - Li]+
[M – Me3N – C18:1 + Li]+
[M – C18:1 + Li] +
C18:1/C18:1-PC
[M + Li]+
[GroP – H2O – H]-
153.1
Figure S1 - Tandem-MS spectrum of C18:1/C18:1-PC (Li+ adduct), the most abundant PC species identified in the positive-ion mode. Fragmentation was performed by total-ion mapping (TIM) using PQD and spectra were analyzed manually. ChoP, phosphatidylcholine; Me3N, trimethylamine. Assigned peaks are indicated. m/z, mass to charge ratio.
