# Supplementary material for: Characterization of Cell Wall Lipids from the Pathogenic Phase of Paracoccidioides brasiliensis Cultivated in the Presence or Absence of Human Plasma
Source: PLoS One. 2013 May 17;8(5):e63372. doi: 10.1371/journal.pone.0063372 (PMC3656940; doi:10.1371/journal.pone.0063372)
Supplement: Figure S2 — Tandem-MS spectrum of C18∶1/C18∶1-PC (HCOO- adduct), the most abundant PC species identified in the negative-ion mode. Fragmentation was performed by TIM using PQD and spectra were analyzed manually. GroP, glycerophosphate; Me, methyl. Assigned peaks are indicated. (PPTX) [file pone.0063372.s002.pptx]

## Slide 1
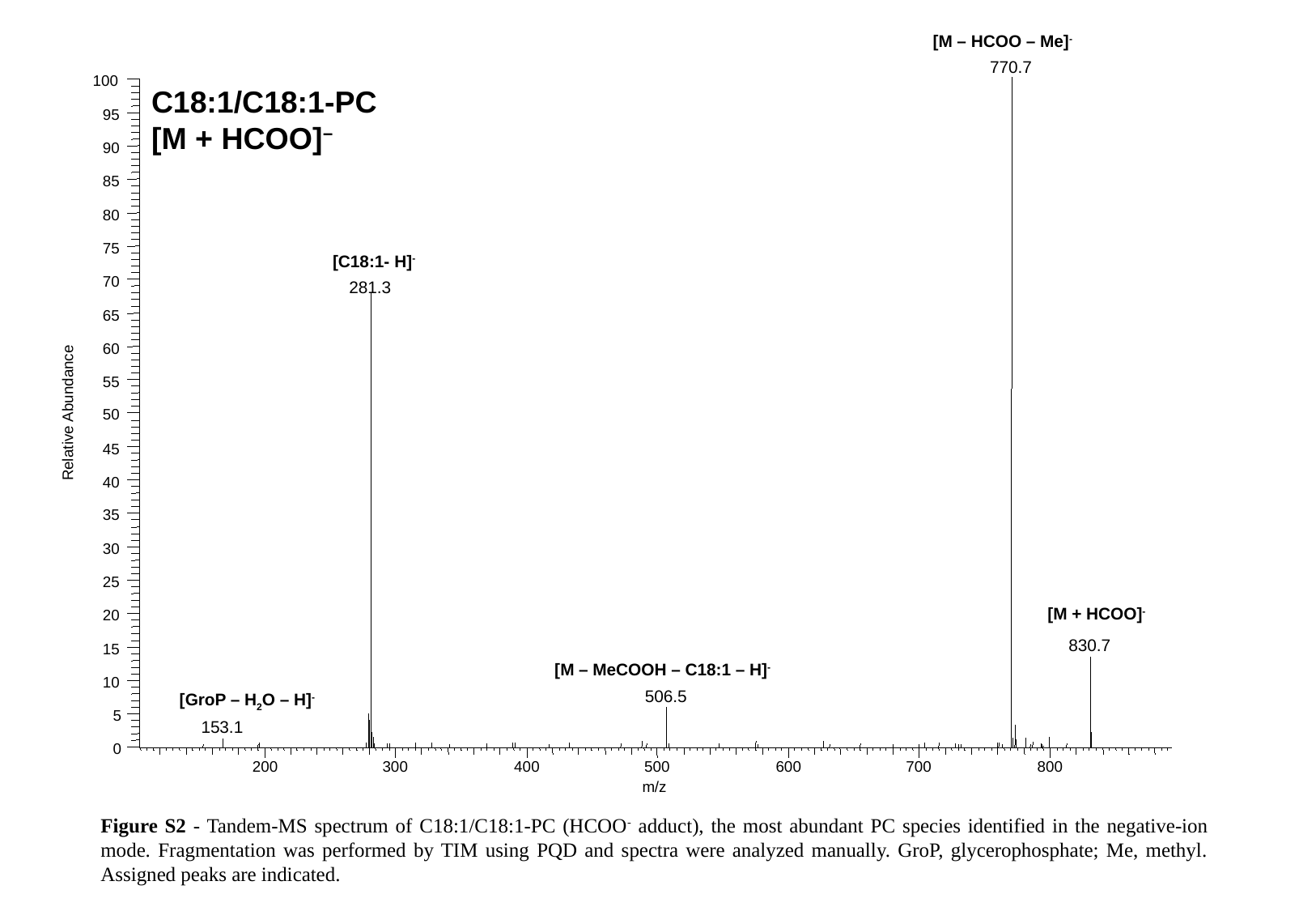

[M – HCOO – Me]-
770.7
100
C18:1/C18:1-PC
[M + HCOO]−
95
90
85
80
75
[C18:1- H]-
70
281.3
65
60
55
Relative Abundance
50
45
40
35
30
25
[M + HCOO]-
20
830.7
15
[M – MeCOOH – C18:1 – H]-
10
[GroP – H2O – H]-
506.5
5
153.1
0
200
300
400
500
600
700
800
m/z
Figure S2 - Tandem-MS spectrum of C18:1/C18:1-PC (HCOO- adduct), the most abundant PC species identified in the negative-ion mode. Fragmentation was performed by TIM using PQD and spectra were analyzed manually. GroP, glycerophosphate; Me, methyl. Assigned peaks are indicated.
