# Supplementary material for: Characterization of Cell Wall Lipids from the Pathogenic Phase of Paracoccidioides brasiliensis Cultivated in the Presence or Absence of Human Plasma
Source: PLoS One. 2013 May 17;8(5):e63372. doi: 10.1371/journal.pone.0063372 (PMC3656940; doi:10.1371/journal.pone.0063372)
Supplement: Figure S3 — Tandem-MS spectrum of C16∶0/C18∶1-PE, the most abundant PE species identified in the negative-ion mode. Fragmentation was performed by TIM using PQD and spectra were analyzed manually. GroP, glycerophosphate. Assigned peaks are indicated. (PPTX) [file pone.0063372.s003.pptx]

## Slide 1
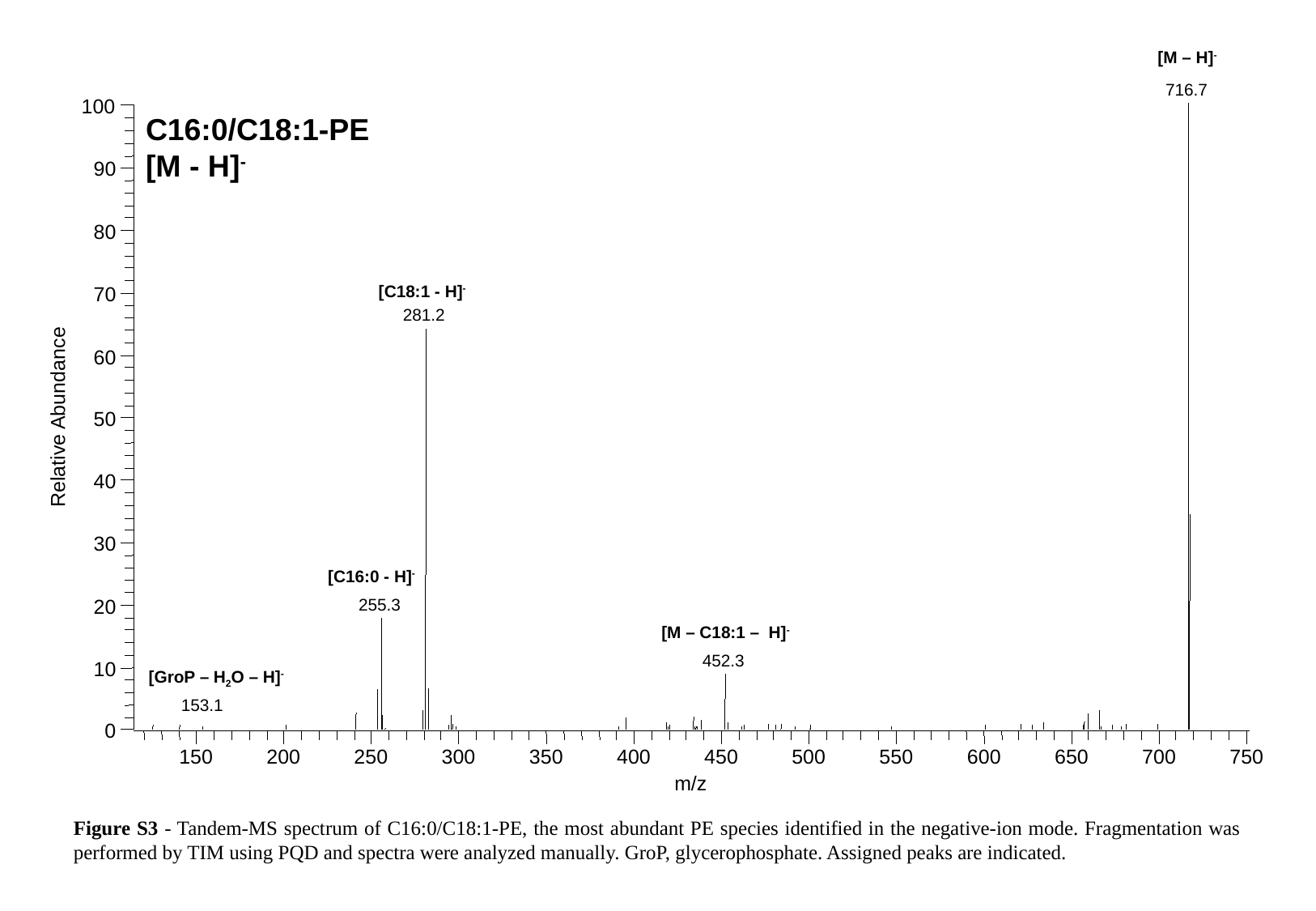

100
90
80
70
60
Relative Abundance
50
40
30
20
10
0
150
200
250
300
350
400
450
500
550
600
650
700
750
m/z
716.7
281.2
255.3
452.3
153.1
[M – H]-
C16:0/C18:1-PE
[M - H]-
[C18:1 - H]-
[C16:0 - H]-
[M – C18:1 – H]-
[GroP – H2O – H]-
Figure S3 - Tandem-MS spectrum of C16:0/C18:1-PE, the most abundant PE species identified in the negative-ion mode. Fragmentation was performed by TIM using PQD and spectra were analyzed manually. GroP, glycerophosphate. Assigned peaks are indicated.
