# Supplementary material for: Characterization of Cell Wall Lipids from the Pathogenic Phase of Paracoccidioides brasiliensis Cultivated in the Presence or Absence of Human Plasma
Source: PLoS One. 2013 May 17;8(5):e63372. doi: 10.1371/journal.pone.0063372 (PMC3656940; doi:10.1371/journal.pone.0063372)
Supplement: Figure S4 — Tandem-MS spectrum of C16∶0/C18∶1-PS, the most abundant PE species identified in the negative-ion mode. Fragmentation was performed by TIM using PQD and spectra were analyzed manually. GroP, glycerophosphate; Ser, serine. Assigned peaks are indicated. (PPTX) [file pone.0063372.s004.pptx]

## Slide 1
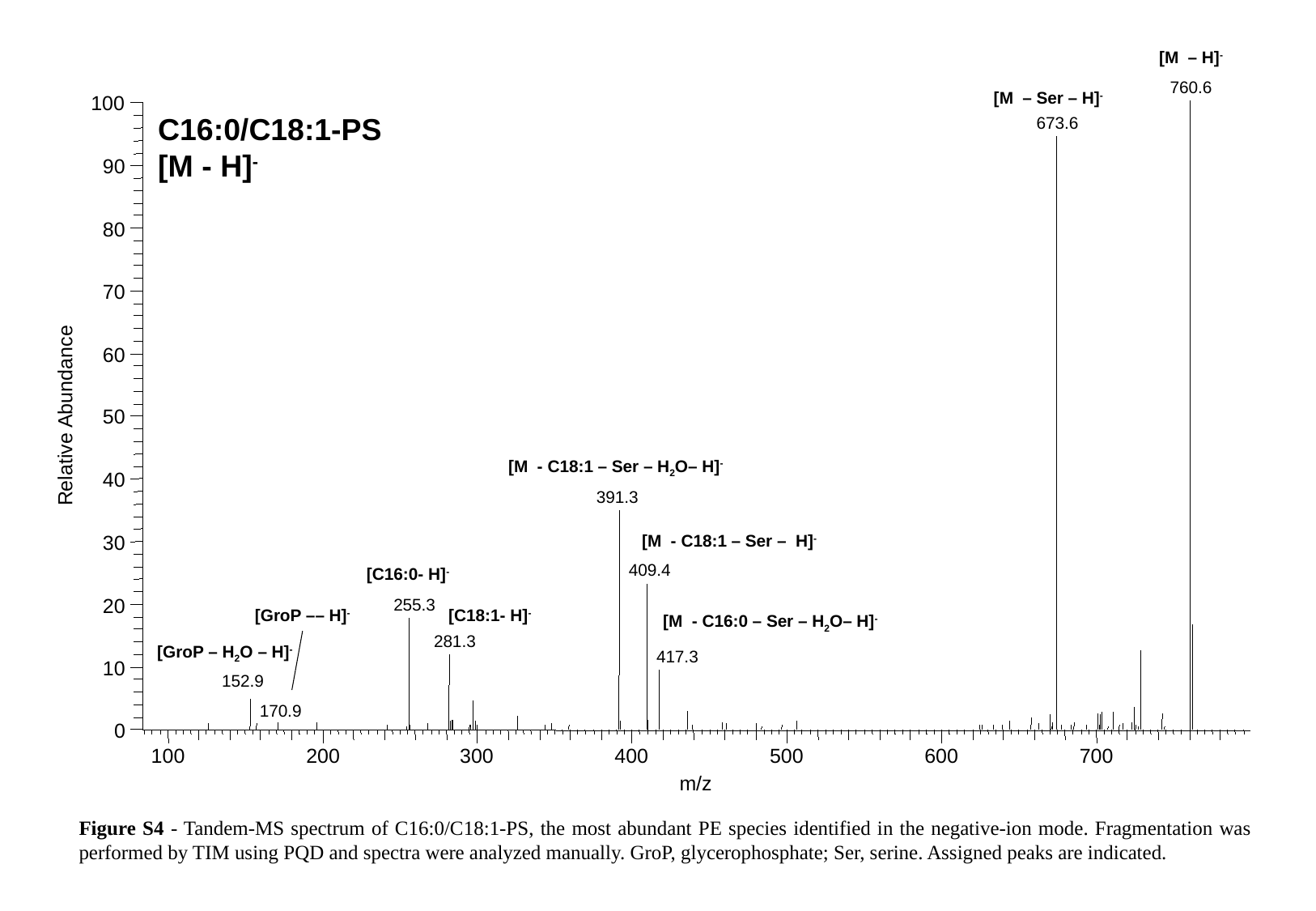

[M – H]-
760.6
[M – Ser – H]-
100
C16:0/C18:1-PS
[M - H]-
673.6
90
80
70
60
Relative Abundance
50
[M - C18:1 – Ser – H2O– H]-
40
391.3
[M - C18:1 – Ser – H]-
30
[C16:0- H]-
409.4
20
255.3
[GroP –– H]-
[C18:1- H]-
[M - C16:0 – Ser – H2O– H]-
281.3
[GroP – H2O – H]-
417.3
10
152.9
170.9
0
100
200
300
400
500
600
700
m/z
Figure S4 - Tandem-MS spectrum of C16:0/C18:1-PS, the most abundant PE species identified in the negative-ion mode. Fragmentation was performed by TIM using PQD and spectra were analyzed manually. GroP, glycerophosphate; Ser, serine. Assigned peaks are indicated.
