# Supplementary material for: Characterization of Cell Wall Lipids from the Pathogenic Phase of Paracoccidioides brasiliensis Cultivated in the Presence or Absence of Human Plasma
Source: PLoS One. 2013 May 17;8(5):e63372. doi: 10.1371/journal.pone.0063372 (PMC3656940; doi:10.1371/journal.pone.0063372)
Supplement: Figure S5 — Tandem-MS spectrum of C16∶0/C18∶2-PI, the most abundant PI species identified in the negative-ion mode. Fragmentation was performed by TIM using PQD and spectra were analyzed manually. GroP, glycerophosphate; Ins, inositol; InsP, phosphoinositol. Assigned peaks are indicated. (PPTX) [file pone.0063372.s005.pptx]

## Slide 1
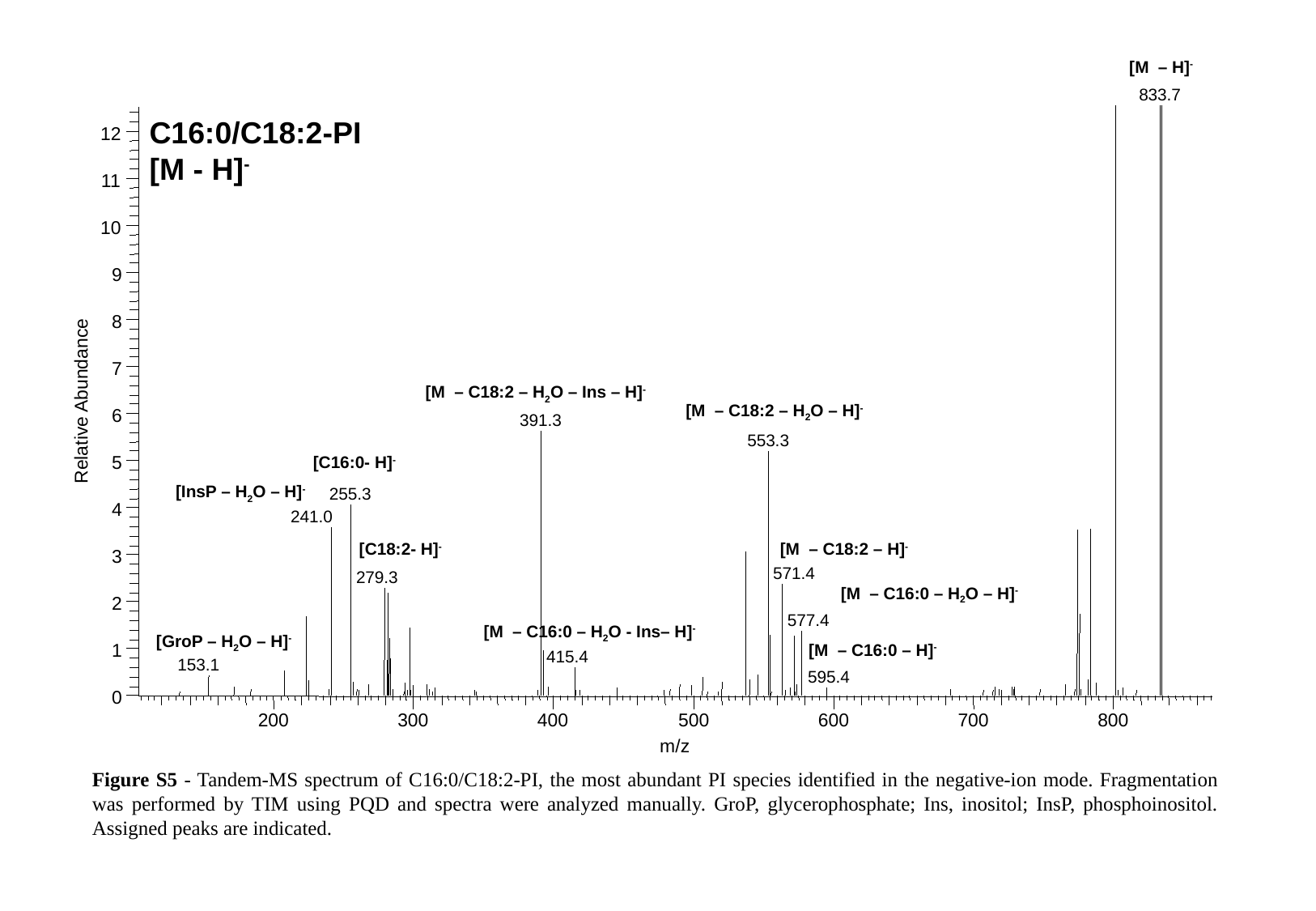

[M – H]-
833.7
C16:0/C18:2-PI
[M - H]-
12
11
10
9
8
7
[M – C18:2 – H2O – Ins – H]-
Relative Abundance
[M – C18:2 – H2O – H]-
6
391.3
553.3
[C16:0- H]-
5
[InsP – H2O – H]-
255.3
4
241.0
[C18:2- H]-
[M – C18:2 – H]-
3
571.4
279.3
[M – C16:0 – H2O – H]-
2
577.4
[M – C16:0 – H2O - Ins– H]-
[GroP – H2O – H]-
[M – C16:0 – H]-
1
415.4
153.1
595.4
0
200
300
400
500
600
700
800
m/z
Figure S5 - Tandem-MS spectrum of C16:0/C18:2-PI, the most abundant PI species identified in the negative-ion mode. Fragmentation was performed by TIM using PQD and spectra were analyzed manually. GroP, glycerophosphate; Ins, inositol; InsP, phosphoinositol. Assigned peaks are indicated.
