# Supplementary material for: Characterization of Cell Wall Lipids from the Pathogenic Phase of Paracoccidioides brasiliensis Cultivated in the Presence or Absence of Human Plasma
Source: PLoS One. 2013 May 17;8(5):e63372. doi: 10.1371/journal.pone.0063372 (PMC3656940; doi:10.1371/journal.pone.0063372)
Supplement: Figure S6 — Tandem-MS spectrum of C16∶0/C18∶1-PG, the most abundant PG species identified in the negative-ion mode. Fragmentation was performed by TIM using PQD and spectra were analyzed manually. GroP, glycerophosphate. Assigned peaks are indicated. (PPTX) [file pone.0063372.s006.pptx]

## Slide 1
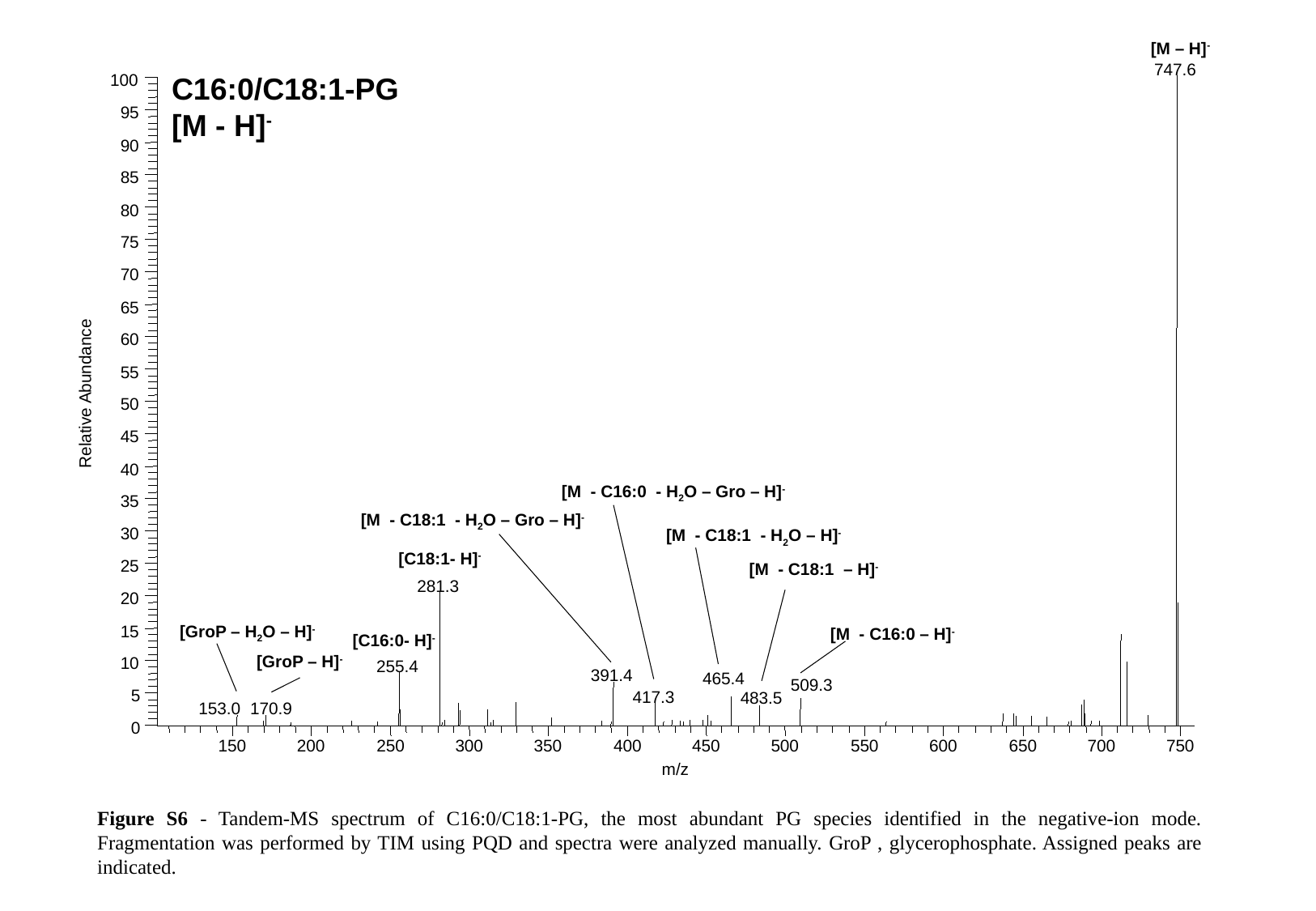

[M – H]-
747.6
C16:0/C18:1-PG
[M - H]-
100
95
90
85
80
75
70
65
60
55
Relative Abundance
50
45
40
[M - C16:0 - H2O – Gro – H]-
35
[M - C18:1 - H2O – Gro – H]-
[M - C18:1 - H2O – H]-
30
[C18:1- H]-
[M - C18:1 – H]-
25
281.3
20
[GroP – H2O – H]-
[M - C16:0 – H]-
15
[C16:0- H]-
[GroP – H]-
10
255.4
391.4
465.4
509.3
483.5
5
417.3
170.9
153.0
0
150
200
250
300
350
400
450
500
550
600
650
700
750
m/z
Figure S6 - Tandem-MS spectrum of C16:0/C18:1-PG, the most abundant PG species identified in the negative-ion mode. Fragmentation was performed by TIM using PQD and spectra were analyzed manually. GroP , glycerophosphate. Assigned peaks are indicated.
