# Supplementary material for: Characterization of Cell Wall Lipids from the Pathogenic Phase of Paracoccidioides brasiliensis Cultivated in the Presence or Absence of Human Plasma
Source: PLoS One. 2013 May 17;8(5):e63372. doi: 10.1371/journal.pone.0063372 (PMC3656940; doi:10.1371/journal.pone.0063372)
Supplement: Figure S7 — Tandem-MS spectrum of 16∶0/18∶2-PA, the most abundant PI acid species identified in the negative-ion mode. Fragmentation was performed by total-ion mapping using PQD and spectra were analyzed manually. GroP, glycerophosphate. Assigned peaks are indicated. (PPTX) [file pone.0063372.s007.pptx]

## Slide 1
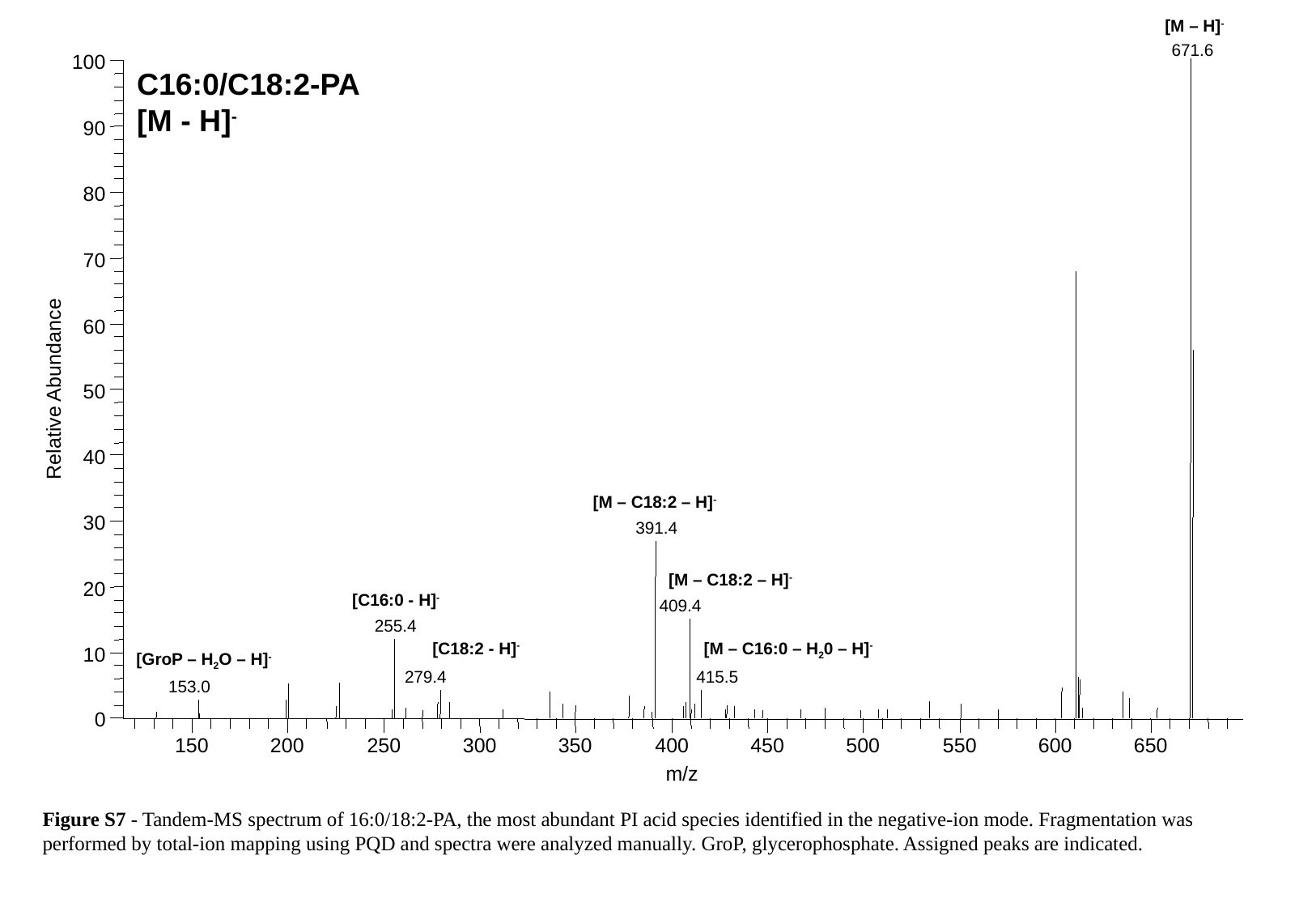

[M – H]-
671.6
C16:0/C18:2-PA
[M - H]-
[M – C18:2 – H]-
391.4
[M – C18:2 – H]-
[C16:0 - H]-
409.4
255.4
[M – C16:0 – H20 – H]-
[C18:2 - H]-
[GroP – H2O – H]-
279.4
415.5
153.0
150
200
250
300
350
400
450
500
550
600
650
m/z
100
90
80
70
60
Relative Abundance
50
40
30
20
10
0
Figure S7 - Tandem-MS spectrum of 16:0/18:2-PA, the most abundant PI acid species identified in the negative-ion mode. Fragmentation was performed by total-ion mapping using PQD and spectra were analyzed manually. GroP, glycerophosphate. Assigned peaks are indicated.
