# Supplementary material for: Characterization of Cell Wall Lipids from the Pathogenic Phase of Paracoccidioides brasiliensis Cultivated in the Presence or Absence of Human Plasma
Source: PLoS One. 2013 May 17;8(5):e63372. doi: 10.1371/journal.pone.0063372 (PMC3656940; doi:10.1371/journal.pone.0063372)
Supplement: Figure S8 — Tandem-MS spectrum of the glycolipid Hex-C16∶0-OH/d19∶2-Cer identified at m/z 848.7. Fragmentation was performed in the positive-ion mode by TIM using PQD and spectra were analyzed manually. Assigned peaks are indicated. (PPTX) [file pone.0063372.s008.pptx]

## Slide 1
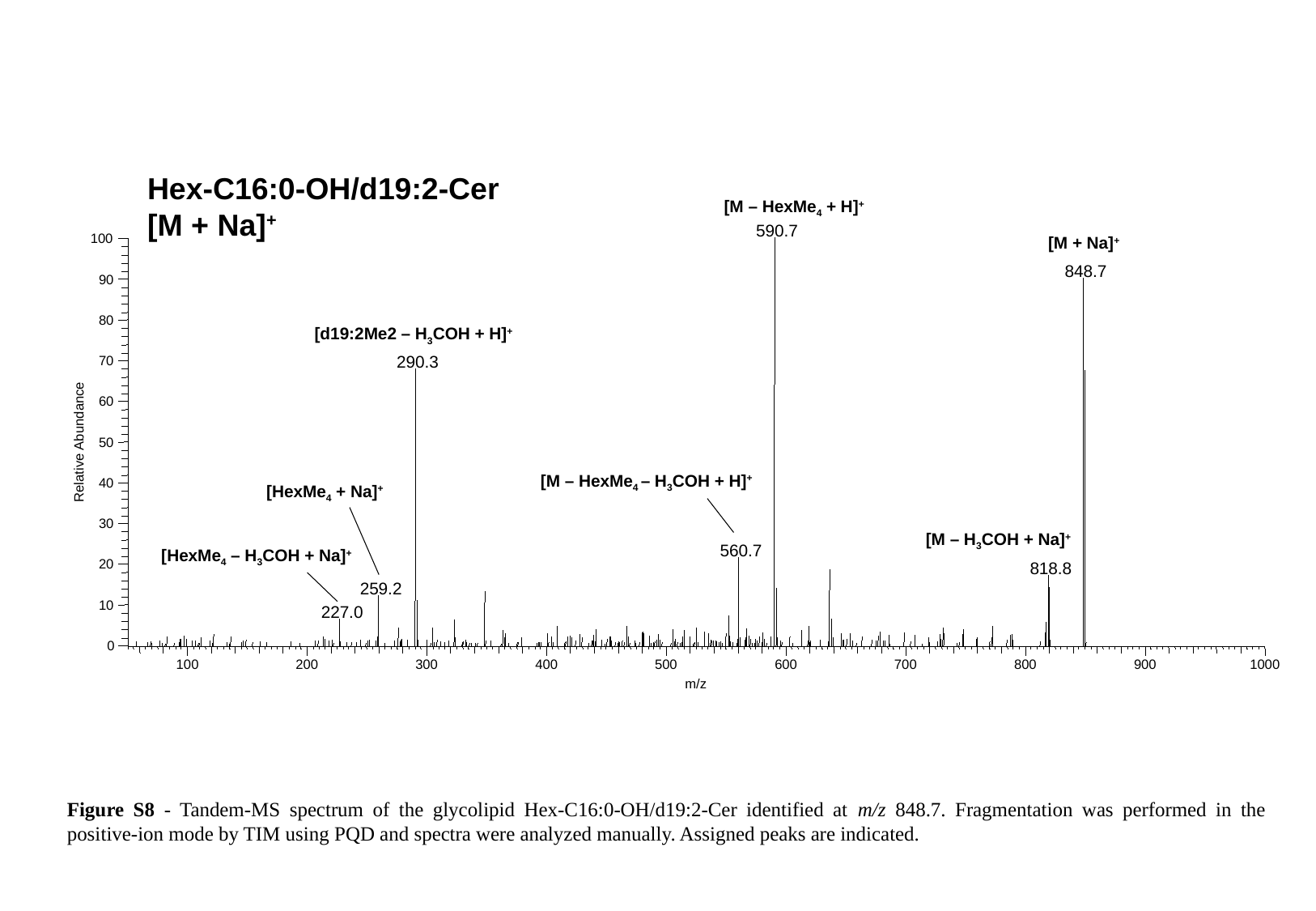

Hex-C16:0-OH/d19:2-Cer
[M + Na]+
[M – HexMe4 + H]+
590.7
[M + Na]+
100
90
80
70
60
Relative Abundance
50
40
30
20
10
0
100
200
300
400
500
600
700
800
900
1000
m/z
848.7
[d19:2Me2 – H3COH + H]+
290.3
[M – HexMe4 – H3COH + H]+
[HexMe4 + Na]+
[M – H3COH + Na]+
[HexMe4 – H3COH + Na]+
560.7
818.8
259.2
227.0
Figure S8 - Tandem-MS spectrum of the glycolipid Hex-C16:0-OH/d19:2-Cer identified at m/z 848.7. Fragmentation was performed in the positive-ion mode by TIM using PQD and spectra were analyzed manually. Assigned peaks are indicated.
