# Supplementary material for: Characterization of Cell Wall Lipids from the Pathogenic Phase of Paracoccidioides brasiliensis Cultivated in the Presence or Absence of Human Plasma
Source: PLoS One. 2013 May 17;8(5):e63372. doi: 10.1371/journal.pone.0063372 (PMC3656940; doi:10.1371/journal.pone.0063372)
Supplement: Figure S9 — Tandem-MS spectrum of the glycolipid Hex-C18∶0-OH/d18∶2-Cer identified at m/z 862.8. Fragmentation was performed in the positive-ion mode by total-ion mapping using pulsed-Q dissociation (PQD) and spectra were analyzed manually. Assigned peaks are indicated. (PPTX) [file pone.0063372.s009.pptx]

## Slide 1
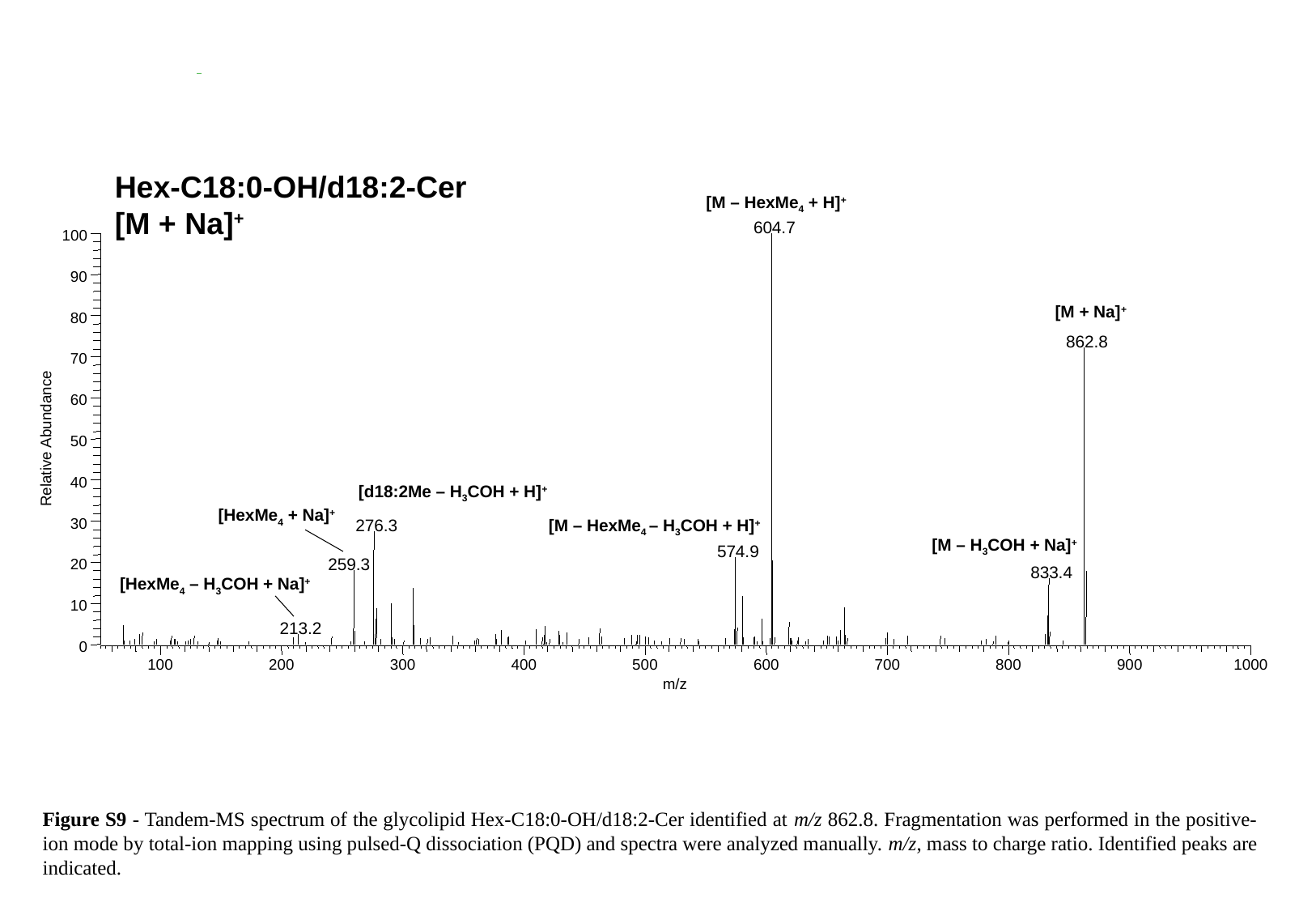

604.7
100
90
80
70
60
Relative Abundance
50
40
30
20
10
0
100
200
300
400
500
600
700
800
900
1000
m/z
862.8
276.3
574.9
259.3
833.4
213.2
Hex-C18:0-OH/d18:2-Cer
[M + Na]+
[M – HexMe4 + H]+
[M + Na]+
[d18:2Me – H3COH + H]+
[HexMe4 + Na]+
[M – HexMe4 – H3COH + H]+
[M – H3COH + Na]+
[HexMe4 – H3COH + Na]+
Figure S9 - Tandem-MS spectrum of the glycolipid Hex-C18:0-OH/d18:2-Cer identified at m/z 862.8. Fragmentation was performed in the positive-ion mode by total-ion mapping using pulsed-Q dissociation (PQD) and spectra were analyzed manually. m/z, mass to charge ratio. Identified peaks are indicated.
