# Supplementary material for: Characterization of Cell Wall Lipids from the Pathogenic Phase of Paracoccidioides brasiliensis Cultivated in the Presence or Absence of Human Plasma
Source: PLoS One. 2013 May 17;8(5):e63372. doi: 10.1371/journal.pone.0063372 (PMC3656940; doi:10.1371/journal.pone.0063372)
Supplement: Figure S10 — Tandem-MS spectrum of the glycolipid Hex-C18∶0-OH/d18∶1-Cer identified at m/z 864.9. Fragmentation was performed in the positive-ion mode by TIM using PQD and spectra were analyzed manually. Assigned peaks are indicated. (PPTX) [file pone.0063372.s010.pptx]

## Slide 1
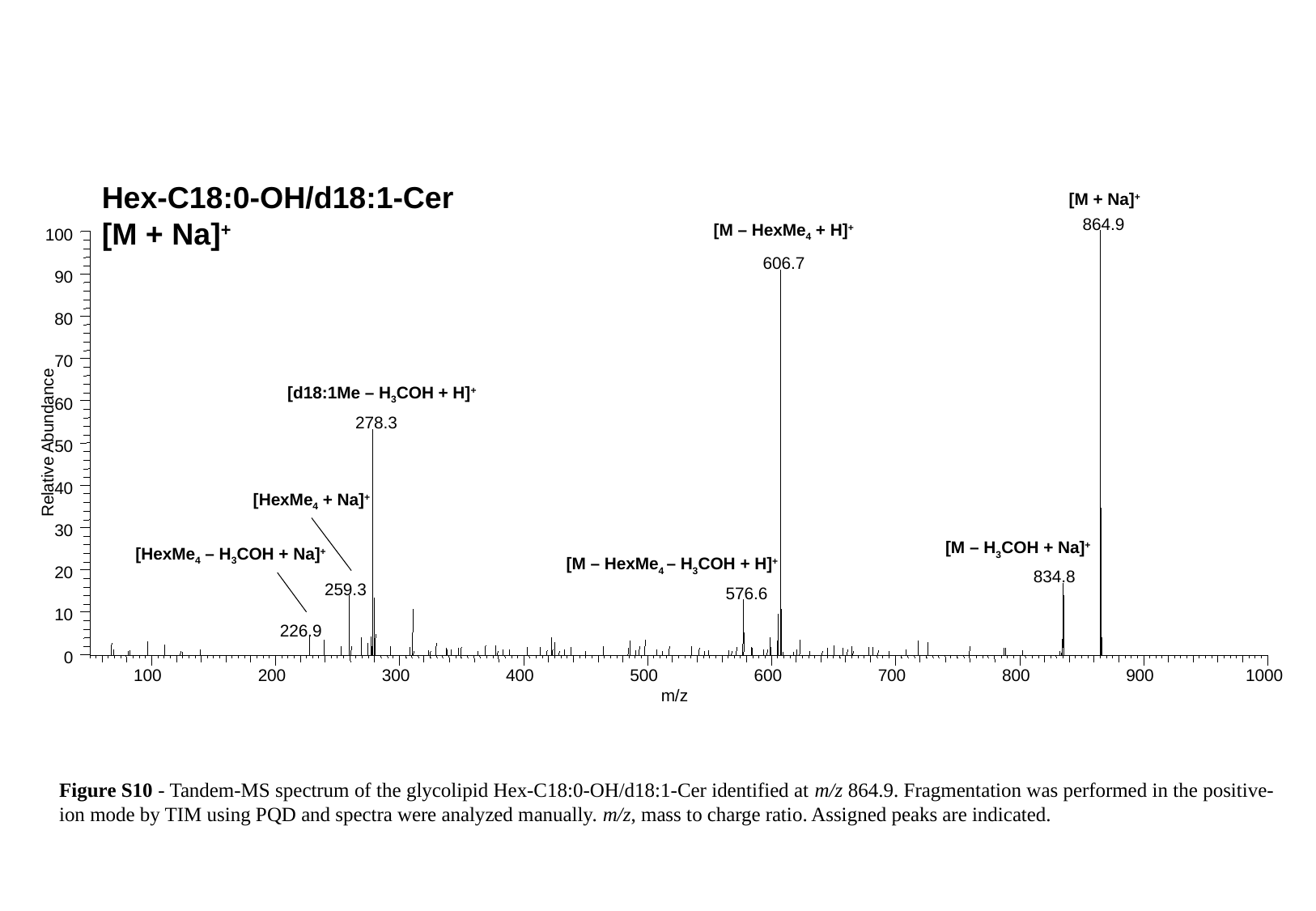

Hex-C18:0-OH/d18:1-Cer
[M + Na]+
[M + Na]+
864.9
100
606.7
90
80
70
60
278.3
Relative Abundance
50
40
30
20
834.8
259.3
576.6
10
226.9
0
100
200
300
400
500
600
700
800
900
1000
m/z
[M – HexMe4 + H]+
[d18:1Me – H3COH + H]+
[HexMe4 + Na]+
[M – H3COH + Na]+
[HexMe4 – H3COH + Na]+
[M – HexMe4 – H3COH + H]+
Figure S10 - Tandem-MS spectrum of the glycolipid Hex-C18:0-OH/d18:1-Cer identified at m/z 864.9. Fragmentation was performed in the positive-ion mode by TIM using PQD and spectra were analyzed manually. m/z, mass to charge ratio. Assigned peaks are indicated.
